# Integrated Analysis of miRNA and mRNA Expression Profiles in Spleen of Specific Pathogen-Free Chicken Infected with Avian Reticuloendotheliosis Virus Strain SNV

**DOI:** 10.3390/ijms20051041

**Published:** 2019-02-27

**Authors:** Shuo Gao, Hao Jiang, Jie Sun, Youxiang Diao, Yi Tang, Jingdong Hu

**Affiliations:** 1 College of Animal Science and Veterinary Medicine, Shandong Agricultural University, No. 61 Daizong Street, Tai’an 271018, Shandong, China; gaoshuo0320@163.com (S.G.); m15610416863@163.com (H.J.); sunjie1230sunjie@163.com (J.S.); yxdiao@163.com (Y.D.); 2 Shandong Provincial Key Laboratory of Animal Biotechnology and Disease Control and Prevention, Shandong Agricultural University, No. 61 Daizong Street, Tai’an 271018, Shandong, China; 3 Shandong Provincial Engineering Technology Research Center of Animal Disease Control and Prevention, Shandong Agricultural University, No. 61 Daizong Street, Tai’an 271018, Shandong, China

**Keywords:** miRNA, mRNA, REV-SNV, SPF chicken, expression profiles, integrated analysis, immunosuppression

## Abstract

The Reticuloendotheliosis virus (REV) primarily causes avian severe immunosuppression, in addition to other symptoms, which include avian dwarfing syndrome and chronic tumors in lymphoid and other tissue. To date, REV’s molecular mechanisms leading to immunosuppression have not been fully elucidated. In the current study, we aimed to elucidate the role of microRNAs (miRNA) in regulating gene expression during REV infections. Therefore, we used a high-dose spleen necrosis virus (SNV) model of REV to inoculate one-day-old specific pathogen-free (SPF) chickens, thereby inducing congenital infections. We analyzed miRNA and mRNA expression profiles using Next Generation Sequencing (NGS) in a total of 19 spleen samples that were collected at 7, 14, and 21 days post infection (dpi). The results showed that 63 differentially expressed miRNAs (DEmiRNAs) (30 known miRNAs and 33 novel miRNAs) and 482 differentially expressed target genes (DETGs) were identified. Integration analysis identified 886 known miRNA–mRNA and 580 novel miRNA–mRNA interaction pairs, which involved miRNAs that were inversely correlated with the above DETGs. Kyoto Encyclopedia of Genes and Genomes (KEGG) enrichment analysis revealed that the DETGs were considerably enriched in the immune-relevant pathways category, such as immune system, cell growth and death, signaling molecules and interaction, signal transduction, etc. We further verified selected immune-relevant miRNA and their DETGs while using quantitative RT-PCR (qRT-PCR). Overall, our data revealed valuable immune-related miRNA–mRNA interaction information that occurred during REV infections, thereby broadening our understanding of the REV-induced immunosuppression.

## 1. Introduction

Reticuloendotheliosis (RE) is a collection of pathological syndromes with the characteristics of acute reticulocyte tumor, dwarf syndrome, formation of chronic tumor in lymphoid and other tissues, and causing severe immunosuppression [1,2]. RE has been reported all over the world since Reticuloendotheliosis virus (REV) was first isolated from turkeys with lymphoma in the 1950s [3]. In China, more than 20 provinces, including Shandong Province, Anhui Province, Yunnan Province, etc., have reported REV infections, with a 20–30% prevalence rate in poultry [4], emphasizing that the direct and indirect hazards to the poultry industry are becoming increasingly serious. RE characteristics, including immunosuppression, tumorigenesis, mixed infection, contaminated vaccine, and genetic recombination, which are harmful to human health, have gradually increased awareness of the disease’s significance [5,6,7].

MicroRNA (miRNA) are endogenous small (22–25 nt in length) non-coding RNAs that are ubiquitously expressed in higher eukaryotic cells. Their role in regulating post-transcriptional gene silencing by controlling mRNA translation into the proteins has been one of the most important life science discoveries in recent years [8,9]. Host cell miRNAs have various functions, such as regulating infecting viral gene expression, interfering with viral replication, and stimulating interferon-mediated antiviral activity. miRNAs have demonstrated a role in triggering inflammation and tumorigenesis, in addition to regulating immune cell differentiation, development, and immune response [10,11].

Due to its nature as a poultry viral tumor disease with immunosuppression as the primary hazard, researching RE has been difficult, due to its clinically inconspicuous pathogenic effect. However, RE’s pathogenic characteristics require further investigation in the context of miRNA-related immune regulation and their mechanisms during viral infection. In recent years, genome-wide transcriptome sequencing using Next Generation Sequencing (NGS) has become effective and inexpensive [12]. Therefore, we established a specific pathogen-free (SPF) chicken artificial infection model using REV-spleen necrosis virus (REV-SNV) [13], and performed NGS on REV-infected spleen tissue cells of SPF chickens, to obtain their mRNA and miRNA expression profiles. We investigated miRNA target gene functions at the level of miRNA regulation. We also integrated miRNA and mRNA expression profiles to preliminarily explore how miRNAs regulate mRNA gene expression during REV infections, to further elucidate the molecular mechanisms of REV-induced immunosuppression.

## 2. Results

### 2.1. Verification of REV Infection in SPF Chickens

**Figure 1:**
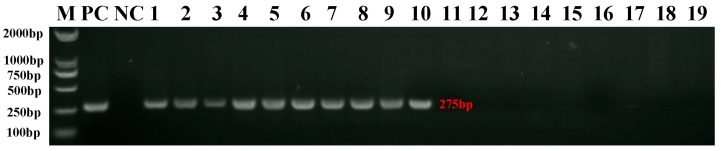
Detection of Reticuloendotheliosis virus-spleen necrosis virus (REV-SNV) viral RNA from infected chicken spleens. The total RNA extracted from virus stock of REV was used as the positive control (PC). The ddH_2_O was used as a negative control (NC). The DNA marker (M) was DL2000 (Takara, Dalian China). Lanes 1–3, 4–6, and 7–10 are the PCR product from the IFN group at 7-, 14-, and 21-dpi, respectively. Lanes 11–13, 14–16, and 17–19 are the PCR product from the CON group at 7-, 14-, and 21-dpi, respectively.

RT-PCR analysis of RNA isolated from infected chicken spleen tissue, followed by agarose gel electrophoresis, validated REV-infection in SPF chickens. The data clearly defined amplified bands at 275 bp in all samples of the infection group, which were then purified and sequenced (Figure 1). The downstream analysis using NCBI/BLAST indicated that the PCR products were identical in sequence to the *LTR* region of REV-SNV (GenBank: DQ003591.1).

### 2.2. Analysis of NGS Data from 19 sRNA Libraries

Nineteen spleen sample libraries from six 7-dpi samples (CON1-1, CON1-2, CON1-3, INF1-1, INF1-2, and INF1-3), six 14-dpi samples (CON2-1, CON2-2, CON2-3, INF2-1, INF2-2, and INF2-3), and seven 21-dpi samples (CON3-1, CON3-2, CON3-3, INF3-1, INF3-2, INF3-3, and INF3-4) groups were generated using NGS. Sequencing data for each library were recorded, as shown in Table 1. Q20 and Q30 values in each library indicated good sequencing quality. Raw data were submitted to the Sequence Read Archive (SRA) of the National Center for Biotechnology Information (NCBI) (https://www.ncbi.nlm.nih.gov/sra/), and the SRA submission ID is SUB4782429, the BioProject ID is PRJNA505870.

**Figure 2:**
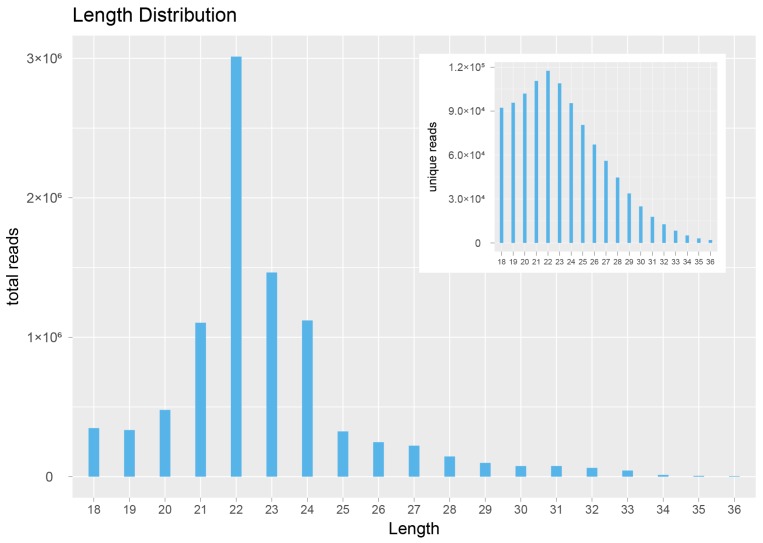
Length distribution results for miRNA sequences. Only INF1-1 sample results are shown. The lower left corner is the total reads result for different lengths, and the upper right corner is the clean reads result for different lengths. The horizontal axis is the length of the sequence, and the vertical axis is sequence abundance (×10,000) of the corresponding length. Read length distribution statistics for the remaining samples are showed in Figure S1.

After raw data processing, we performed statistical analysis on clean/total reads with sequence lengths above 18 nt before and after deduplication (Figure 2). The results showed that, by majority, the clean/total reads were 21–24 nt long, with 22 nt miRNAs being the most abundant.

**Figure 3:**
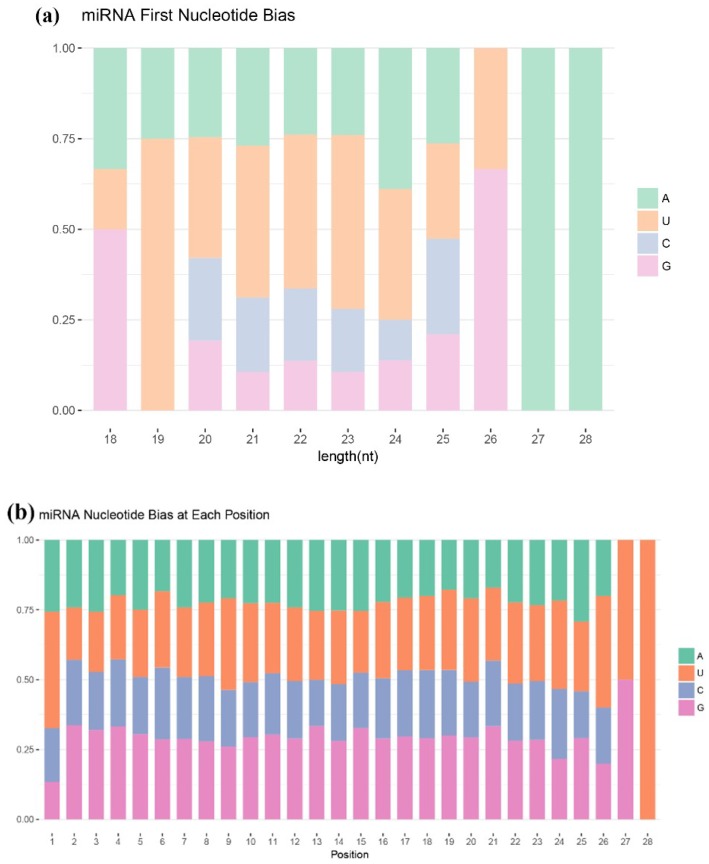
miRNA nucleotide bias analysis results. (**a**) Results of miRNA first nucleotide bias; and, (**b**) results of miRNA nucleotide bias at each position.

miRNA nucleotide bias in different nucleotide positions was determined by calculating the occurrence frequency of the first nucleotide for miRNAs with different lengths (Figure 3a), and the occurrence frequency of each nucleotide of all the miRNAs (Figure 3b). The results showed that A was the first base for 27–28 nt long miRNAs, G for 18–26 nt long miRNA, and U for 19–25 nt long miRNA. Moreover, the miRNAs had the strongest preference for U at position 28, and U or G at position 27. The base preferences were roughly equivalent at other positions.

### 2.3. Gene Expression Analysis and Target Prediction

**Figure 4:**
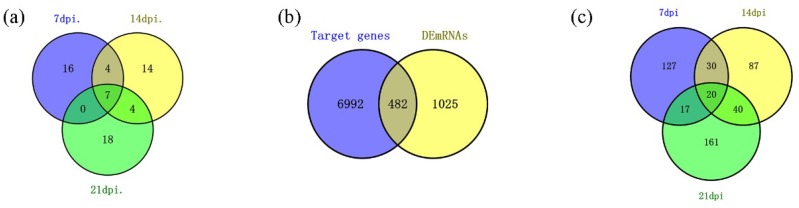
Venn diagrams. (**a**) The number and overlap of DEmiRNAs in spleens infected with REV at 7-, 14-, and 21-dpi. Venn chart analysis identified seven miRNAs (six known and one novel miRNAs) that were up-regulated at all three tested time points. (**b**) Target genes represent the candidate target genes (CTGs) of 7373 differentially expressed miRNAs predicted using TargetScan. DEmRNAs represent 1507 differentially expressed mRNAs. We identified 482 differentially expressed target genes (DETGs) using a Venn chart. (**c**) The number and overlap of DETGs in spleens infected with REV at 7-, 14-, and 21-dpi. Venn chart analysis identified 20 DETGs (17 up- and three down-regulated) that were always differentially expressed at all three tested time points.

**Figure 5:**
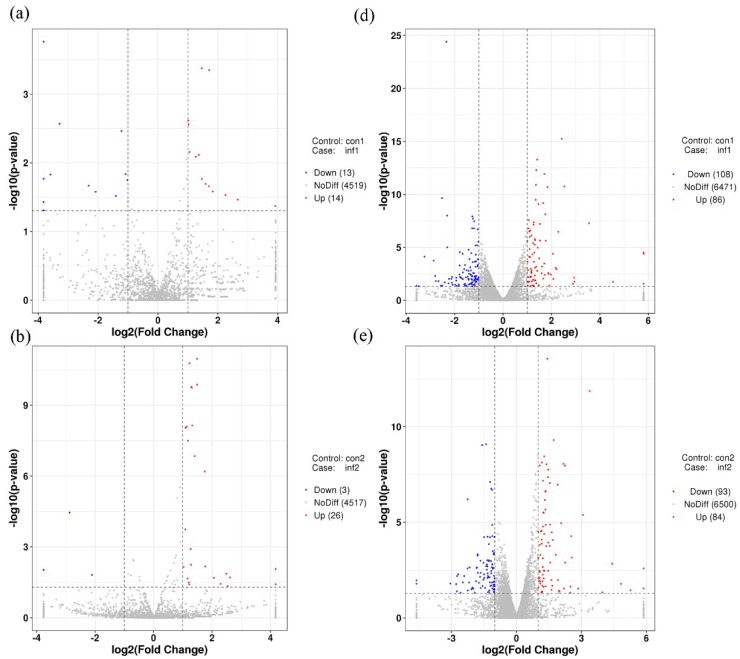

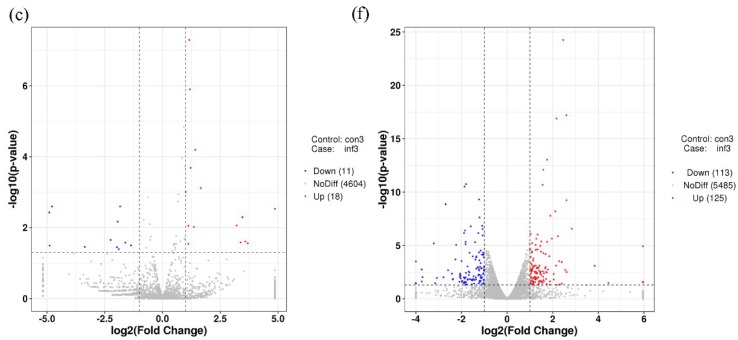
Volcano chart of miRNA (**a**–**c**) and CTG (**d**–**f**) expression in the 19 libraries. The *x*-axis shows the Log2 (fold change) and *y*-axis shows the −log10 (q-value). Red points represent the upregulated miRNAs/CTG and green points represent the downregulated miRNAs/CTG. The vertical line in the figure is a two-fold difference threshold; the horizontal line is a *p* < 0.05 threshold.

The final data were obtained through normalization and statistical analysis using DESeq (version 1.18.0) to identify miRNAs that are associated with REV infection in SPF chickens. We identified 63 miRNAs that were differentially expressed between groups CON and INF on 7-, 14-, and 21-dpi. Among these 63 miRNAs (Figure 4a), 27 were identified in 7-dpi samples, with 13 and 14 that were down- and up-regulated, respectively (Figure 5a). Twenty-nine were identified in 14-dpi samples, three and 26 were down- and up-regulated, respectively (Figure 5b). In addition, 29 miRNAs were identified in 21-dpi samples, with 11 and 18 down- and up-regulated, respectively (Figure 5c).

We identified a total of 7373 candidate target genes (CTGs) that were generated by TargetScan prediction, of which 63 differentially expressed miRNAs (DEmiRNAs) and 482 differentially expressed target genes (DETGs) were identified by intersection analysis, with 1507 differentially expressed mRNAs (DEmRNAs) (Figure 4b). Among these 482 DETGs (Figure 4c): 194 were identified in 7-dpi samples, 108 and 80 of which were down- and up-regulated, respectively (Figure 5d); 177 were identified in 14-dpi samples, 93 and 84 of which were down- and up-regulated, respectively (Figure 5e); and, 238 DETGs were identified in 21-dpi samples, with 113 and 125 being down- and up-regulated, respectively (Figure 5f).

### 2.4. Functional Enrichment Analysis of DEmiRNA

**Figure 6:**
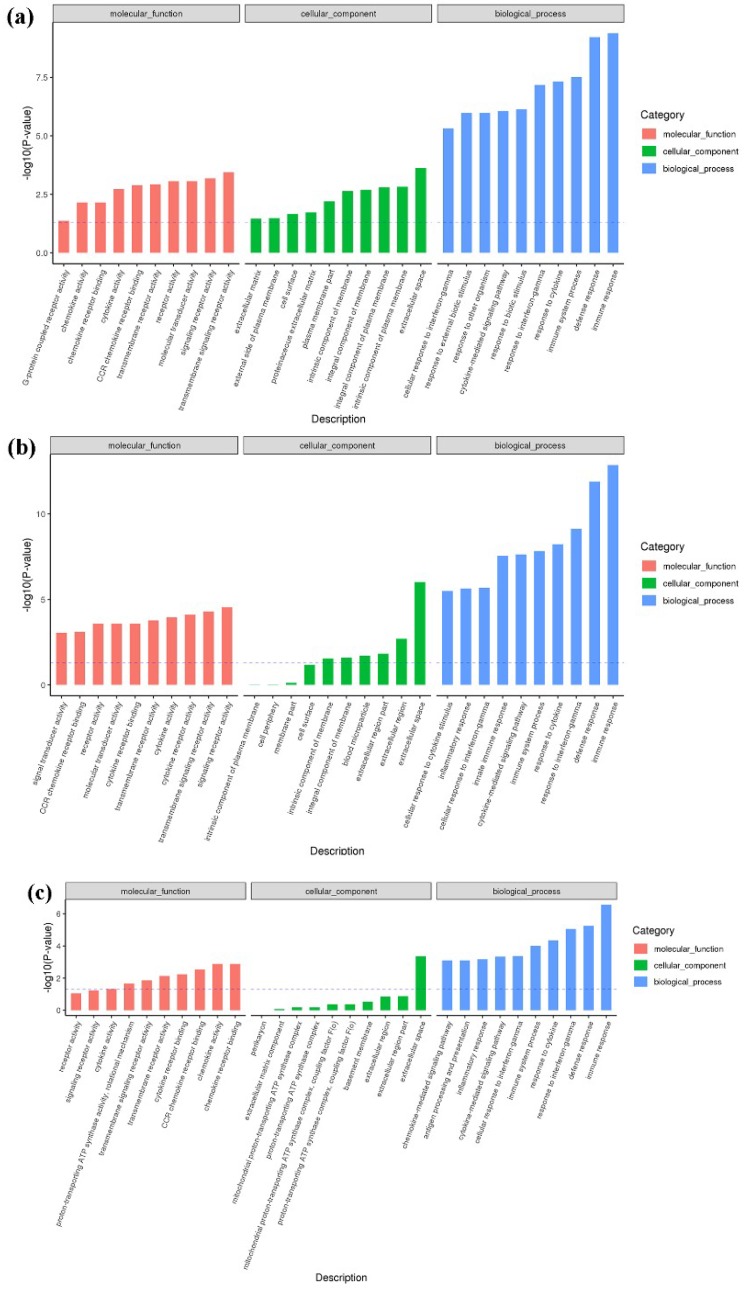
CTG functional enrichment analysis at 7- (**a**), 14- (**b**), and 21-dpi (**c**). Each color represents a different biological process. The *x*-axis indicates the description and the *y*-axis indicates the -log10 (*p*-value). Only the top 10 Gene Ontology (GO) terms are listed in this figure.

To further understand the potential functions of miRNA in host chicken infected with REV, and to explore the distribution and potential biological functions of CTGs, we analyzed the data using Gene Ontology (GO) functional enrichment analysis. The analysis entailed identifying significantly enriched GO functional terms of the CTGs of DEmiRNAs at 7-, 14-, and 21-dpi. As shown in Figure 6, CTGs were mainly related to biological regulation and cellular essential components at 7-, 14-, and 21-dpi. In addition, the unique functions of DEmiRNA CTGs at 7-dpi primarily included localization, regulation of signaling, tissue development, and intracellular transport (Figure 6a). Unique functions of DEmiRNA CTGs at 14-dpi mainly included RNA metabolic process, embryonic organ development, and positive regulation of neurogenesis (Figure 6b). Unique DEmiRNA CTG functions at 21-dpi mainly included protein metabolic process, intracellular signal transduction, glycoprotein biosynthetic process, and cardiac septum development (Figure 6c). Details of the GO terms are included in Table S1.

**Figure 7:**
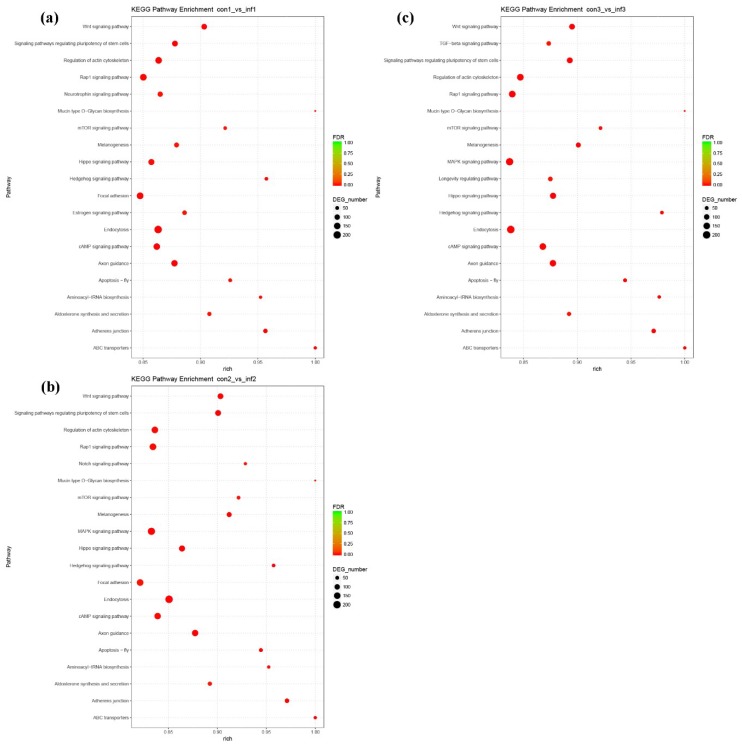
Kyoto Encyclopedia of Genes and Genomes (KEGG) pathway analysis of CTGs at 7- (**a**), 14- (**b**), and 21-dpi (**c**). The *x*-axis and *y*-axis show enrichment and pathway names, respectively. Point size represents the number of genes.

We then applied Kyoto Encyclopedia of Genes and Genomes (KEGG) enrichment analysis to further understand the potential miRNA REV-infected host chicken biological, metabolic, and signaling pathways of CTGs. The analysis revealed the main DEmiRNA CTG pathways at 7-, 14-, and 21-dpi. As shown in Figure 7, the CTGs were mainly enriched in the following categories: signal transduction, glycan biosynthesis and metabolism, immune system, endocrine system and cellular community/cell growth, and death at 7-, 14-, and 21-dpi. The immune-related signal pathways were Wnt signaling pathway, cAMP signaling pathway, Rap1 signaling pathway, Hippo signaling pathway, mTOR signaling pathway, MAPK signaling pathway, ABC transporters, Apoptosis, and Endocytosis. Moreover, a complete list of descriptions for CTGs that are involved in pathways is shown in the Table S2.

### 2.5. miRNA–mRNA Integrated Analysis

Comprehensive analysis of interacting DEmiRNA and DEmRNA can reveal valuable information regarding the role of DEmiRNAs in REV infections. In this analysis, we focused on inversely-related DEmiRNA–DEmRNA pairs, i.e., down-regulated miRNAs matching up-regulated mRNAs, or up-regulated miRNAs matching the down-regulated mRNAs. We predicted a total of 680 and 206 different interaction pairs, which involved 20 known up-regulated miRNAs that matched 206 down-regulated mRNAs (Table S3), and 10 known down-regulated miRNAs that matched 102 up-regulated mRNAs (Table S4), respectively. In addition, 580 inverse correlation pairs of novel DEmiRNAs and DEmRNAs were predicted (Table S5). The results showed that most of the miRNAs had many target genes. For example, *gga-miR-1329-5p* was inversely correlated with 22 genes, *gga-miR-21* and *gga-miR-222b* were inversely correlated with 21 genes, and *gga-miR-146b* was inversely correlated with 16 genes. Most mRNAs were associated with more than one miRNA: *STAT1* was targeted by *gga-miR-34b/c*, *gga-miR-375/122*, and *gga-miR-1664*; *CASP10* was targeted by *gga-miR-1618-5p* and *gga-miR-1664-3p*; and, *MAPK10* was targeted by *gga-miR-146b-3p*, *gga-miR-147*, and *gga-miR-222b-5p*.

**Figure 8:**
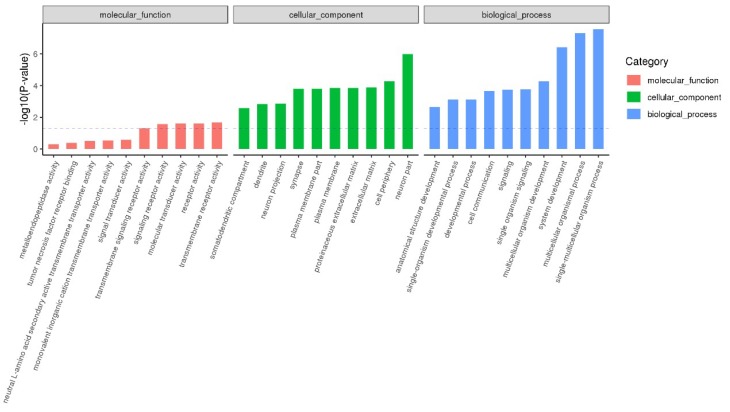
Functional enrichment analysis of DETGs. Each color represents a different biological process. The *x*-axis shows the description and the *y*-axis is the −log10 (*p*-value). Only the top 10 GO terms are listed.

GO functional enrichment analysis identified significantly enriched GO functional terms that are associated with DETGs. As shown in Figure 8, REV infection was mainly related to cellular organism processes, biological regulation, membrane components, receptor activity, and cellular components. More detailed data on DETG-related GO analysis are shown in Table S6.

**Figure 9:**
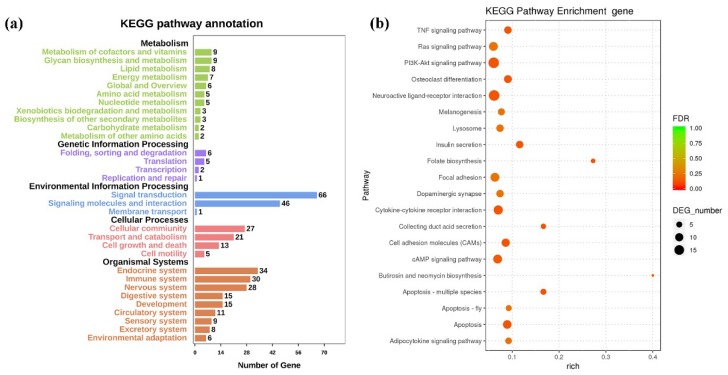
(**a**) KEGG pathway annotation and (**b**) analysis of DETGs.

**Figure 10:**
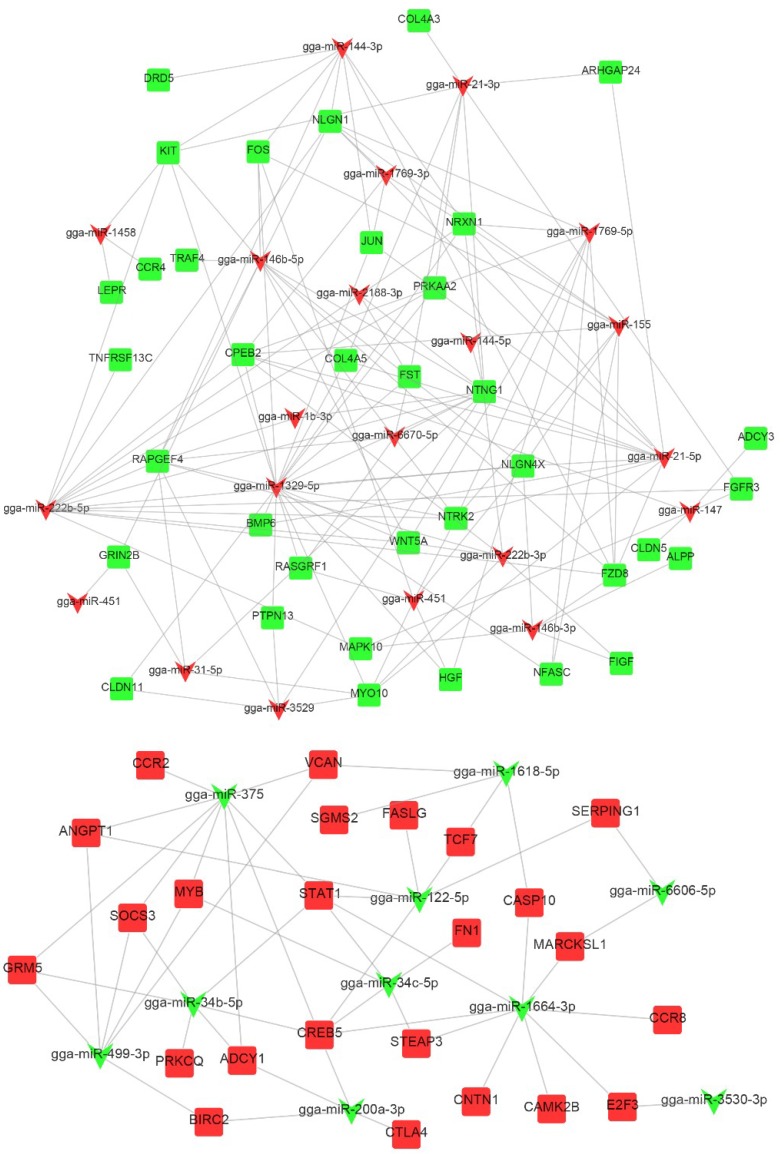
Networks diagram between miRNAs and inversely correlated target genes that are involved in immunity. Rectangular boxes represent genes and V-shapes represent miRNAs that target those genes. Red and green represent genes that are up- and down-regulated, respectively.

KEGG enrichment analysis was performed to further explore DETGs functions following a REV infection. As shown in Figure 9a, the KEGG functional terms contained a large number of enriched DETGs, which are mainly involved in the following categories: immune system, signal transduction, signaling molecules and interaction, endocrine system, nervous system, cellular community, transport, and catabolism. DETGs were mainly enriched in signal pathways, including PI3K-Akt signaling pathway, cytokine-cytokine receptor interaction, cAMP signaling pathway, focal adhesion, ras signaling pathway, apoptosis, Rap1 signaling pathway, cell adhesion molecules (CAMs), TNF signaling pathway, ECM-receptor interaction, etc. (Figure 9b). A complete list describing DETGs that are involved in pathways is shown in Table S7. The negative correlation between known miRNA and mRNA expression in the categories of cell growth and death, signaling molecules and interaction, signal transduction, and immune system were visualized while using Cytoscape (Figure 10).

### 2.6. Validation of DEmiRNAs and Targets by Quantitative RT-PCR (qRT-PCR)

**Figure 11:**
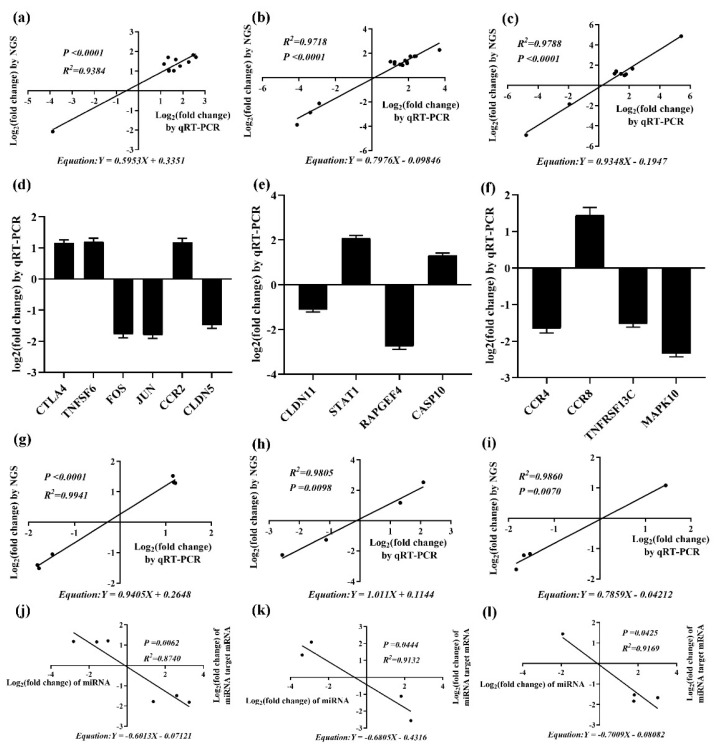
Validation of DEmiRNAs and their targets by quantitative RT-PCR (qRT-PCR). (**a**–**c**) are correlation analyses of miRNA expression at 7-, 14-, and 21-dpi, respectively. The *x*-axis shows the Log2 (fold change) by qRT-PCR, and the *y*-axis is the Log2 (fold change) by NGS. (**d**–**f**) are expression levels of the above target mRNAs at 7-, 14-, and 21-dpi, respectively (the *y*-axis shows the Log2 (fold change) by qRT-PCR. (**g**–**i**) are correlation analyses of the above target mRNAs expression at 7-, 14-, and 21-dpi, respectively. The *x*-axis shows the Log2 (fold change) by qRT-PCR, and the *y*-axis is the Log2 (fold change) by NGS. (**j**–**l**) are qRT-PCR results of miRNAs and their target mRNAs at 7-, 14-, and 21-dpi, respectively (the *x*-axis shows the Log2 (fold change) of miRNAs, and the *y*-axis is the Log2 (fold change) of their target mRNAs).

To validate the NGS data, qRT-PCR was used to verify 15 miRNAs, which were generated at the intersection of any two time points at 7-, 14-, and 21-dpi. The 15 miRNAs include seven miRNAs which were differentially expressed at both 7-, 14-, and 21-dpi (*gga-miR-147*, *gga-novel-3165-5p*, *gga-miR-1329-5p*, *gga-miR-21-5p*, *gga-miR-222b-5p*, *gga-miR-146b-3p*, and *gga-miR-222b-3p*); four miRNAs that were only differentially expressed at 7- and 14-dpi (*gga-miR-144-3p*, *gga-miR-451*, *gga-miR-146b-5p*, and *gga-miR-1618-5p*); and four miRNAs which were only differentially expressed at 14- and 21-dpi (*gga-miR-21-3p*, *gga-miR-1664-3p*, *gga-novel-5516-5p*, and *gga-novel-0698-5p*). Gene expression changes of the 15 DEmiRNAs were comparable between the three time points, with correlation coefficients of R^2^ = 0.9384 (*p* < 0.0001), 0.9718 (*p* < 0.0001), and 0.9788 (*p* < 0.0001) (Figure 11a–c). The qRT-PCR results were consistent with the sequencing results, indicating that the NGS results were reliable for differential gene identification. Some minor differences still exist in the details between the two methods, although their outputs were similar in the trend of gene expression change. This is largely due to the procedural and parametric differences between the two technologies, but it has little impact on the overall study.

Based on functional enrichment analysis and clinical symptoms of RE immunosuppression, we selected immune-related target genes *CTLA4*, *CCR2*, *CCR4*, *CCR8*, *TNFSF6*, *TNFRSF13C*, *CLDN5*, *CLDN11*, *CASP10*, *STAT1*, *MAPK10*, *RAPGEF4*, *FOS*, and *JUN* for validation. These target genes were regulated by *gga-miR-200a-3p*, *gga-miR-375*, *gga-miR-1458*, *gga-miR-1664-3p*, *gga-miR-122-5p*, *gga-miR-222b-5p*, *gga-miR-147*, *gga-miR-1329-5p*, *gga-miR-1618-5p*, *gga-miR-1664-3p*, *gga-miR-146b-3p*, *gga-miR-222b-3p*, *gga-miR-144-3p*, and *gga-miR-1769-3p*, respectively. As shown in Figure 11d–f, the expression of *CTLA4*, *TNFSF6*, *CCR2*, *CCR8*, *STAT1*, and *CASP10* were significantly up-regulated, whereas those of *FOS*, *JUN*, *CLDN5*, *CLDN11*, *RAPGEF4*, *CCR4*, *TNFRSF13C*, and *MAPK10* were significantly down-regulated. In addition, the qRT-PCR results of those target genes were also consistent with the NGS results, with correlation coefficients of R^2^ = 0.9941 (*p* < 0.0001), 0.9805 (*p* = 0.0098), and 0.9860 (*p* = 0.0070) (Figure 11g–i). In addition, the expression of miRNAs and their DETGs were negatively correlated (R^2^ = 0.8740, 0.9132, and 0.9169; *p* = 0.0062, 0.0444, and 0.0425) (Figure 11j–l).

## 3. Discussion

miRNA regulation represents a new dimension throughout regulatory networks and it plays a key role in different regulatory pathways, including developmental time control, cell differentiation, proliferation, apoptosis, metabolism, hormone secretion, tumorigenesis, immune response, as well as other physiological and pathological processes [8,9]. In recent years, many scholars have carried out research on host–virus interaction that is based on miRNA levels in the field of medical and virological research [14]. Studies using the avian leukemia (AL) and Marek’s disease (MD) model for host and viral miRNA expression changes have also been widely reported [15,16,17,18,19,20,21,22,23]. REV is classified as a mammalian C-type retrovirus, and it is immunologically, morphologically, and structurally distinct from the avian leukemia/sarcoma virus (ALV) population [24]. REVs that have been isolated thus far have similar antigenicity and only one serotype. Nevertheless, RE-induced chickens have various tumor syndromes that are similar to MD and AL. General and histological lesions are also very similar. REVs also have a broader host range when compared to other avian neoplastic viruses, can infect and transform a variety of cells, and have the most immunosuppressive effect [25,26,27]. Therefore, RE can be used as a good model for studying chicken-specific immunosuppressive diseases [28,29]. At the same time, RE can be used as a model for studying lymphocytic diseases and other retrovirally-induced tumors.

Ji Miao used microarrays to analyze the transcriptome of CEF and found that immune response-related genes play an important role in the pathogenicity of REV infections [30]. Zhiqiang Yu et al. identified the effect of REV infections on miRNA expression profile changes in the bursa of SPF chickens, and found that miRNAs are not only involved in up-regulating pro-apoptotic, proto-oncogene, and cancer cell-associated genes, but also down-regulating genes that are associated with anti-apoptotic cytokines [31]. Jie Zhai et al. identified functional miRNAs in REV-infected CEF cells using transcriptome sequencing [32]. Defang Zhou et al. demonstrated that REV and ALV-J synergistically increased the accumulation of exosomal miRNAs, shedding light on the synergistic molecular mechanism of ALV-J and REV [33]. However, to the best of our knowledge, there have been no reports of integrated analyses of miRNA and mRNA expression profiles in hosts that are infected with REV. In this study, REV exhibits pathogenic characteristics of a higher immunosuppressive effect for chickens at a young age. In chickens, the spleen is an important peripheral immune organ, a place for colonization, and immune response of mature T and B lymphocytes. The main pathogenicity of the REV strain SNV is concentrated in the spleen. Therefore, we integrated the spleen mRNA and miRNA expression profile analysis after SPF chicken infection with REV-SNV at 7-, 14-, and 21-dpi.

In the present study, 27, 29, and 29 DEmiRNAs were identified on 7-, 14-, and 21-dpi, respectively. We analyzed the possible functions of target genes that are regulated by DEmiRNAs. Our results showed that these DEmiRNAs are involved in a variety of biological pathways, such as signal transduction, endocrine system, immune system, cell growth and death, etc. These results revealed that the REV-related changes in miRNAs levels regulate multiple biological functions, and we used DEmiRNA and DEmRNA integration to identify the functional miRNA target relationships. In this part, we focused on the miRNA–mRNA pairs with an inverse correlation. By observing changes in mRNA and miRNA at the same time point, we identified 886 known miRNA–mRNA interaction pairs and 580 novel miRNA–mRNA interaction pairs. KEGG pathway analyses showed that mRNAs that were negatively correlated with miRNAs were involved in multiple signaling pathways in the immune system category (such as Chemokine signaling pathway, Toll-like receptor signaling pathway, NOD-like receptor signaling pathway, T cell receptor signaling pathway, RIG-I-like receptor signaling pathway, etc.), suggesting an important regulatory role for miRNAs. For example, *CTLA4*, which negatively correlated with *gga-miR-200a-3p*, was a negative regulator of T cell activation [34]. *TNFRSF13C*, which negatively correlated with *gga-miR-222b-5p*, was involved in the survival and maturation of B cells [35]. The down-regulation of *gga-miR-200a-3p* and up-regulation of *gga-miR-222b-5p* caused a decrease in the number of immune effector cells that may be contributing to immunosuppression by decreasing the number of immune cells that are available to combat REV infections. In addition, lymphocyte apoptosis may contribute to immunosuppression by mechanisms other than apoptotic cells to actively suppress inflammatory responses [36]. *AP-1* (*Fos*/*Jun*), which was negatively correlated with *gga-miR-144-3p* and *gga-miR-1329-5p*, is a positive modulator of apoptosis [37]. *TNFSF6*, which negatively correlated with *gga-miR-122-5p*, is an initiator of apoptosis in lymphoid and nonlymphoid tissues, which is mediated by binding to its receptor [38]. The down-regulation of *gga-miR-122-5p* and up-regulation of *gga-miR-144-3p* and *gga-miR-1329-5p* may play pivotal roles in the suppression of the host immune response. *EPAC2* (*RAPGEF4*), which was negatively correlated with *gga-miR-222b*, *gga-miR-146b-5p*, and *gga-miR-144-3p*, acted as a novel cAMP mediator in the regulation of innate and adaptive immune cell functions [39]. Some scholars have speculated regarding the role that *EPAC2* may play in monocyctes and macrophages, integrin-mediated adhesion, as well as T and B lymphocytes [40]. *MAPK10* (*JNK3*), which negatively correlated with *gga-miR-222b-5p*, *gga-miR-147*, and *gga-miR-146b-3p*, plays an active role in preserving cells from cytokine attacks and suppressing tumorigenesis [41,42]. However, REV-induced miRNA upregulation (*gga-miR-222b*, *gga-miR-146b*, *gga-miR-147*, and *gga-miR-144-3p*) may result in increased apoptosis and tumorigenesis.

## 4. Materials and Methods

### 4.1. Ethics Statements

All of the animal infection experiments were conducted in accordance with the guidelines of international, national, and institutional guidelines. All of the chicks were maintained in negative-pressure-filtered air isolators and fed as recommended. The animal procedures were performed according to the guidelines of the Committee on Ethics of Animals of Shandong and the appropriate biosecurity, and the Animal Care and Use Committee of Shandong Agricultural University approved the protocol (No. SDAUA-2018-165, February 2018).

### 4.2. REV Infections and Sample Collection

The REV strain SNV (GenBank: DQ003591.1) was graciously provided by Professor Shuhong Sun and Zhizhong Cui from Shandong Agricultural University [13]. All of the SPF White Leghorn line chickens were purchased from the Jinan SAIS Poultry Co. Ltd., China. On the first experimental day, 24 SPF chickens were randomly divided into two groups: the control group (group CON) and the REV-infected group (group INF). Group INF was inoculated with 200 μL containing 5000 tissue culture infectious doses (TCID50s) by intraperitoneal injection at day 1 of age. The CON group did not receive any treatment. Three SPF chickens per group were euthanized on 7-, 14-, and 21-dpi, and the spleen was collected, which were then immediately frozen in liquid nitrogen and stored at −80 °C for later use.

### 4.3. Total RNA Isolation and Virus Infection Assays

Total RNA was extracted from spleen sample collected at 7-, 14-, and 21-dpi using miRNA Purification Kit (CWBIO, Beijing, China), according to the manufacturer’s instructions. Each spleen sample was weighed and 20 mg was used for standardization. A spectrophotometer determined the concentration and purity of total RNA (DS 11, DeNovix, Wilmington, DE, USA.). Total RNA integrity was determined using an Agilent 2100 Bioanalyzer (Agilent, Palo Alto, CA, USA). The concentration of high-quality RNA samples (i.e., A260/280 was between 1.8–2.2, A260/230 ≥ 2.0, and RNA integrity numbers ≥ 7) was normalized to 500 ng/μL, and then used in subsequent experiments.

The sequence of the REV strain SNV (GenBank: DQ003591.1) is published on NCBI. Primers were designed using Oligo 7.0 software [43], and the sequences of the two primers were: 5′-ATCCAATCACGAGCAAACACG-3′ *LTR* forward and 5′-GCCAGCCTACACCACGAACAAAAT-3′ *LTR* reverse, which can amplify the section of long terminal repeat (*LTR*) to verify REV-infection in *chickens*. The PrimeScript™ One Step RT-PCR Kit Ver.2 (Dye Plus; Takara, Dalian, China) was used to amplify the section of *LTR*, under the following conditions: initial denaturation at 94 °C for 3 min, followed by 32 cycles of denaturation at 94 °C for 30 s, annealing at 57 °C for 30 s, and extension at 72 °C for 30 s, with a final extension for 3 min at 72 °C. TSINGKE Biololgical Technology (Beijing, China) sequenced the PCR products.

### 4.4. Library Construction and NGS

The small RNA library were constructed using the NEBNext Multiplex Small RNA Library Prep Set for Illumina (New England Biolabs, Inc., Ipswich MA, UK), according to the manufacturer’s instructions. Briefly, 1μg of total RNA from spleen samples was ligated to a 3′ adapter and a 5′ adapter using the Ligation Enzyme Mix. The resulting samples were reverse-transcribed using Superscript II reverse transcriptase. Enrichment and purification of libraries were done by PCR and agarose gel electrophoresis, respectively. Small RNA libraries were analyzed for QC and the average size of inserts was approximately 140 to 150 bp. The library was quality tested by Agilent High Sensitivity DNA Kit (Agilent Technologies Inc., Nanjing, China) using an Agilent 2100 Bioanalyzer (Agilent, Palo Alto, CA, USA), and the final libraries were quantified using the Quant-iT PicoGreen dsDNA Assay Kit (Thermo Fisher Scientific, Waltham, MA, USA). Partially overlapping primer-based PCR (POP-PCR) amplification and sequencing using a single-strand library as a template on the Illumina HiSeq 2500 sequencing platform using NGS technology was performed based on the standard protocols at Shanghai Personal Biotechnology Co., Ltd.

### 4.5. Analysis of sRNA Sequencing Data

Raw data of the small RNA with low-quality sequences were trimmed using Cutadapt (version 1.2.1) to obtain a clean reading. The Q20 and Q30 of the raw data were also calculated. Subsequently, a clean read number with a sequence length greater than 18 nt was calculated and a unique reading was obtained by deleting the exact same sequence in each sample (OmicShare tools) (www.omicshare.com/tools). MiRdeep2 mapped the selected clean reads to the *gallus* reference genome (ftp://ftp.ensembl.org/pub/release-81/fasta/gallus_gallus/dna/) [44] in order to avoid errors, such as substitutions, insertions, deletions, etc. Being generated during the sequencing process, the mature miRNA and miRNA precursor sequences of *gallus* in miRBase (http://www.mirbase.org/) were then mapped to obtain small RNA annotations. The special hairpin structure of the miRNA precursor was used to predict novel miRNAs using the mireap software (https://sourceforge.net/projects/mireap/). The reads of each known and novel miRNAs were normalized using transcripts per kilobase million (TPM) [45]. Normalized expression = (mapped readcount × 1,000,000)/libsize (libsize: total miRNA readcounts), and the significance was determined by normalizing the raw reads and calculating the *p*-value while using DESeq (http://bioconductor.org/packages/release/bioc/html/DESeq.html), miRNA with |fold change| > 1 and *p*-value < 0.05 were identified as DEmiRNAs [46].

### 4.6. Integrative Analyses of DEmiRNAs and DEmRNAs

To determine the DEmiRNA biological functions, we targeted the 3′UTR sequence of the *gallus* using TargetScan that continuously updated the miRNA target prediction database for target gene prediction of DEmiRNAs. Subsequently, DEmRNAs identified DEmiRNA CTGs. If the CTGs showed a significantly altered mRNA expression, then those genes were identified as DETGs of DEmiRNAs. In addition, GO enrichment analysis, which was performed using GOslim and KEGG analyses, was employed to discover DETGs and CTGs of DEmiRNAs, and analyze the signaling pathways that are involved [47,48]. Cytoscape analysis was performed using the OmicShare tools (www.omicshare.com/tools).

### 4.7. The Expression of miRNAs and Their Targets was Validated by qRT-PCR

To validate the NGS data, qRT-PCR was used to confirm the expression levels of several modulated miRNAs. The Mir-X™ miRNA First-Strand Synthesis Kit (TaKaRa, Dalian, China) synthesized the miRNA’s first-strand cDNA. The U6 snRNA was used as the internal reference gene. For mRNA quantification, the HiFiScript gDNA Removal cDNA Synthesis Kit (CWBIO, China) was used to synthesize first-strand cDNA. *GAPDH* was used as the internal reference gene. The relative expression values were normalized by the internal control. Quantitative real-time PCR analysis was performed using LightCycler^®^96 (Roche Diagnostics GmbH, Germany) with TB Green™ Premix Ex Taq™ II (Tli RNaseH Plus) Kit (Takara, Dalian, China), following the manufacturer’s instructions. Triplicate qRT-PCR was performed on each cDNA sample to guarantee the reproducibility of the amplification. After amplification, the relative fold change of the differentially expressed miRNAs and mRNA were calculated through the 2^−ΔΔ*C*T^ method [49]. The primers were designed using the Oligo 7.0 software and they are listed in Table S8. TSINGKE Biololgical Technology (China) synthesized all of the primers used in this study.

### 4.8. Statistical Analyses

All data are shown as the mean ± standard deviation of three replicates. We performed the correlation analysis of Log2 (fold change) by NGS Sequencing and Log2 (fold change) by qRT-PCR with the software GraphPad Prism, Version 7.0.

## 5. Conclusions

In summary, we successfully established and used the avian RE model to identify DEmiRNAs in the early stage of REV infection. By inversely correlating miRNA expression, the miRNA–mRNA interaction pairs were predicted. This is the first report to integrate miRNA and mRNA expression data in SPF chickens that were infected with REV, which will help to further elucidate the molecular mechanism of REV-induced immunosuppression.

## Figures and Tables

**Table 1:** Sequencing data generated using an Illumina HiSeqTM 2500 sequencer.

Sample	Raw Read	Clean Reads (≥18 nt)	Mature miRNAs	miRNA Precusors	Q20(%)	Q30(%)
con1-1	21,210,076	16,264,810	476	374	95.46	92.91
con1-2	32,179,715	18,018,370	484	384	95.46	92.92
con1-3	11,032,471	7,661,330	401	313	95.48	92.03
inf1-1	10,502,004	9,185,246	428	343	95.07	93.05
inf1-2	10,628,615	7,875,010	389	315	95.37	92.62
inf1-3	22,783,772	15,997,877	486	389	95.57	92.01
con2-1	17,989,084	14138332	444	350	96.66	92.34
con2-2	14,349,826	11,647,721	416	338	96.70	92.37
con2-3	12,940,512	10,611,534	408	324	96.39	92.81
inf2-1	16,402,412	13,810,002	429	340	95.03	93.98
inf2-2	16,231,649	13,895,082	434	347	94.10	92.29
inf2-3	16,781,428	14,092,339	433	339	94.36	92.65
con3-1	12,330,618	10,047,514	413	325	95.74	92.34
con3-2	11,178,524	9,091,076	403	315	95.82	92.53
con3-3	9,816,714	8,692,449	425	336	95.55	93.25
inf3-1	16,059,318	13,727,564	438	350	96.43	92.85
inf3-2	15,587,419	13,357,222	430	341	96.54	92.13
inf3-3	15,102,237	12,748,493	434	343	96.57	92.17
inf3-4	10,884,563	9,182,693	397	317	96.72	92.44

## Supplementary Materials

Supplementary materials can be found at https://www.mdpi.com/1422-0067/20/5/1041/s1.
