# Supplementary figures and images for: Integrated Analysis of miRNA and mRNA Expression Profiles in Spleen of Specific Pathogen-Free Chicken Infected with Avian Reticuloendotheliosis Virus Strain SNV
Source: Int J Mol Sci. 2019 Feb 27;20(5):1041. doi: 10.3390/ijms20051041 (PMC6429403; doi:10.3390/ijms20051041)

# Length Distribution

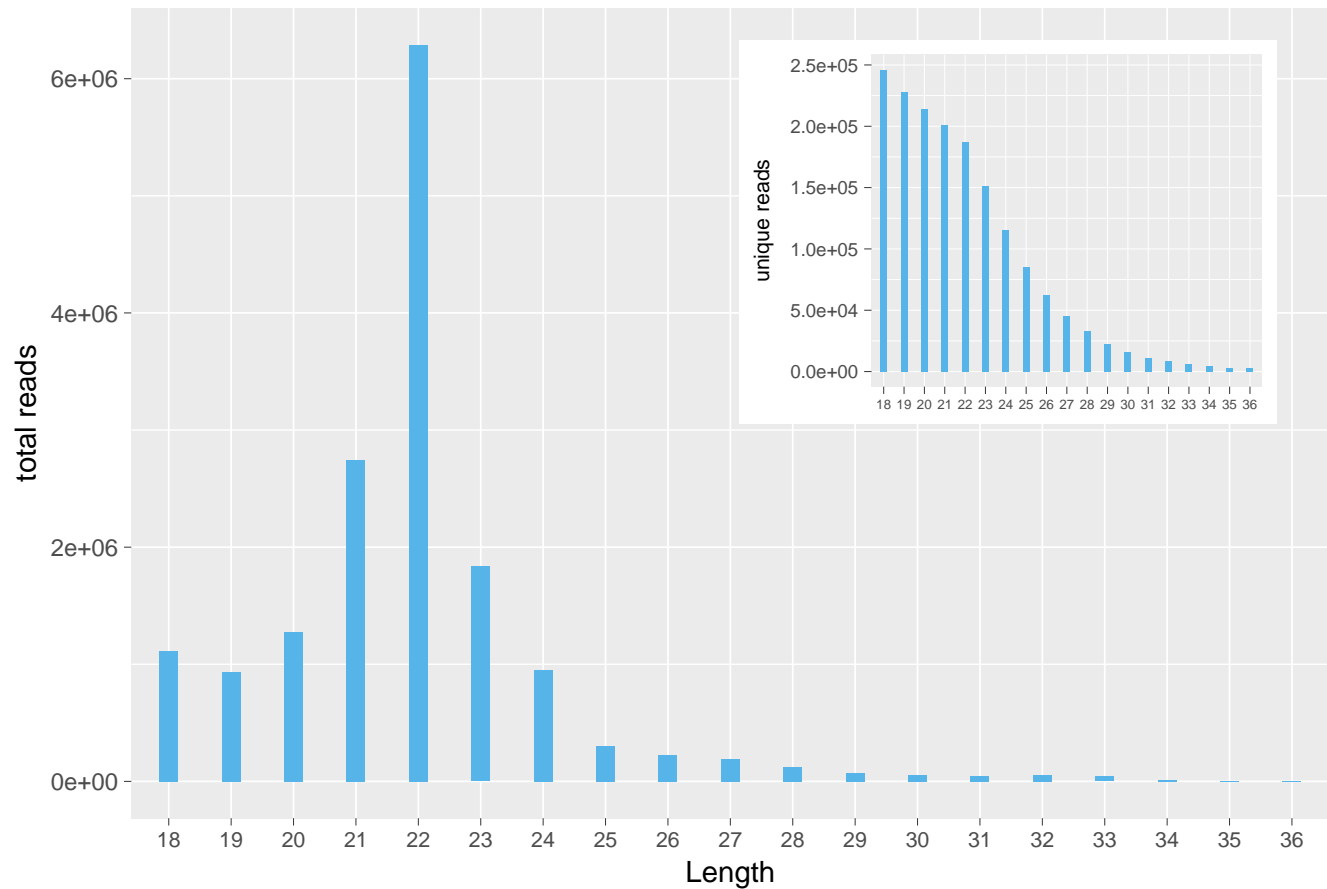

Supplement: Supplementary file 1 [file ijms-20-01041-s001.zip › supplement/Figure S1/con1-1_length_distribution.pdf]

# Length Distribution

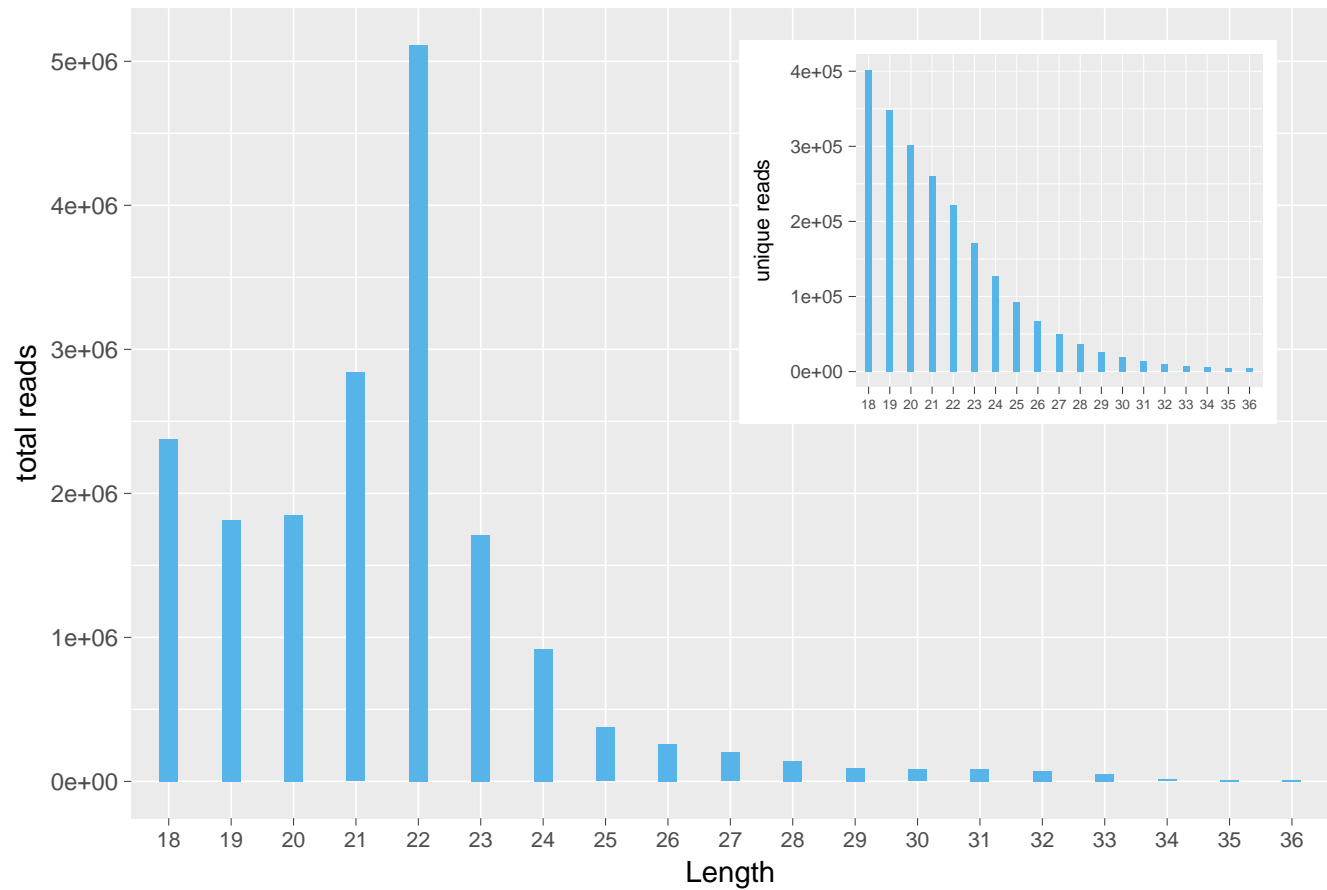

Supplement: Supplementary file 1 [file ijms-20-01041-s001.zip › supplement/Figure S1/con1-2_length_distribution.pdf]

# Length Distribution

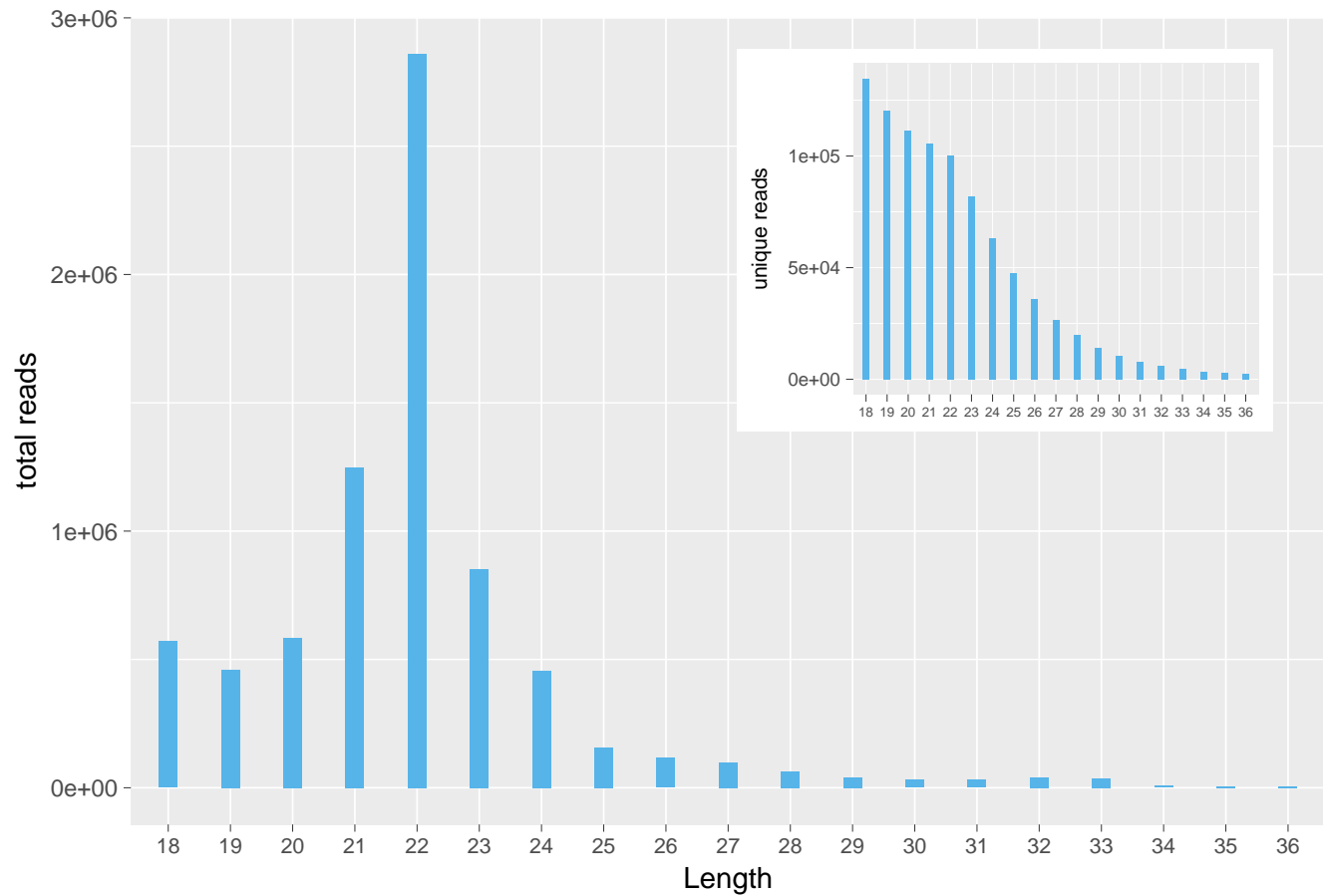

Supplement: Supplementary file 1 [file ijms-20-01041-s001.zip › supplement/Figure S1/con1-3_length_distribution.pdf]

# Length Distribution

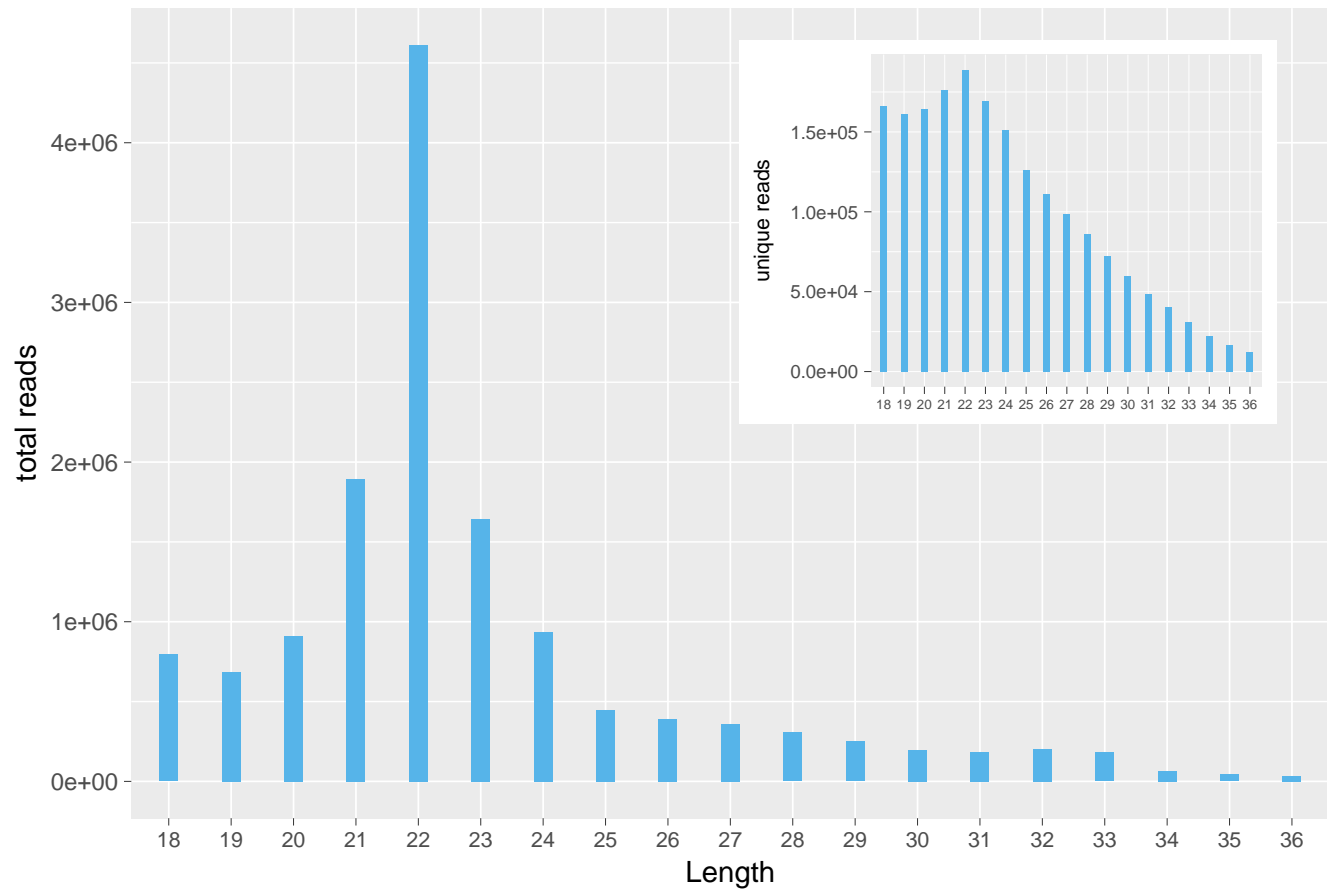

Supplement: Supplementary file 1 [file ijms-20-01041-s001.zip › supplement/Figure S1/con2-1_length_distribution.pdf]

# Length Distribution

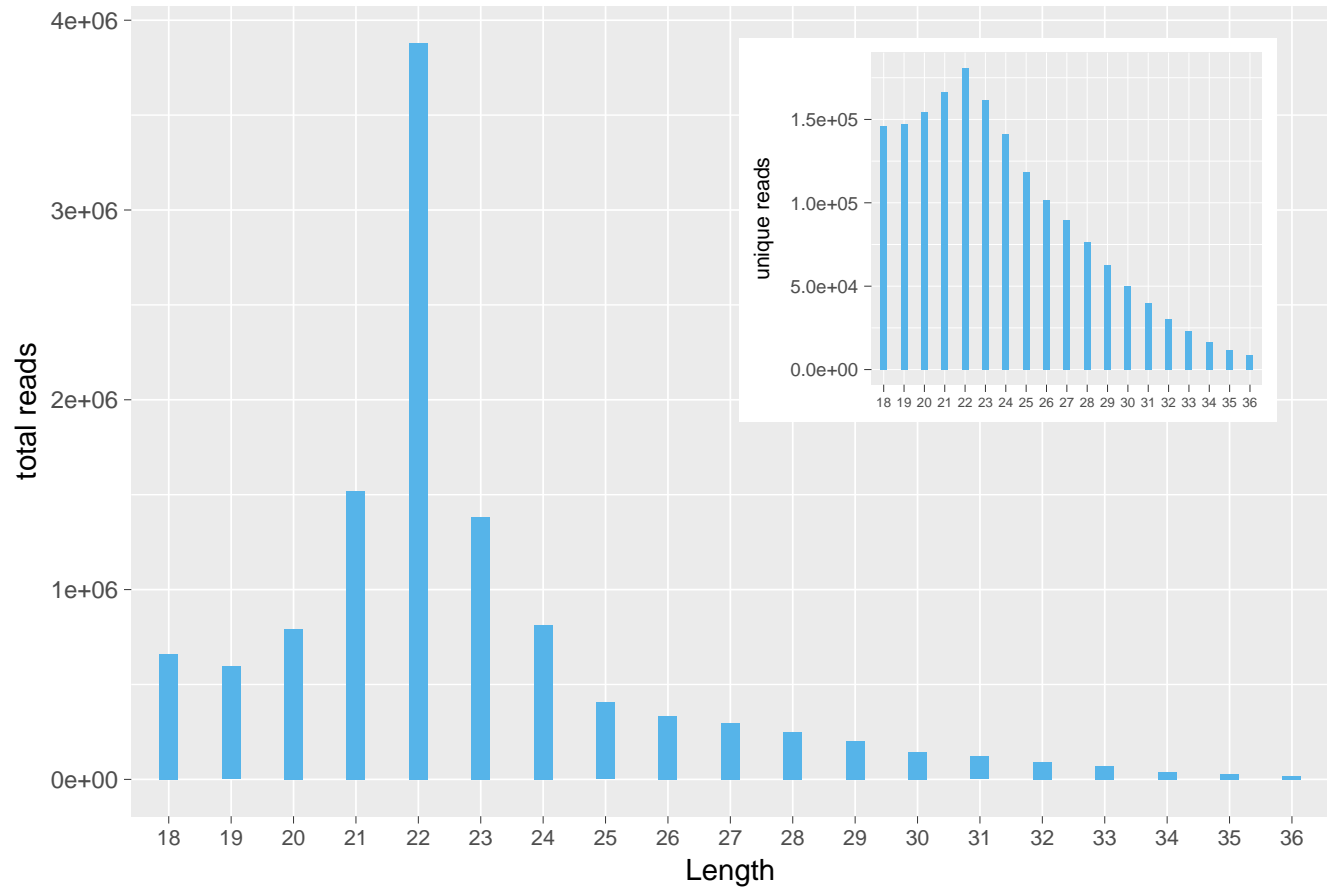

Supplement: Supplementary file 1 [file ijms-20-01041-s001.zip › supplement/Figure S1/con2-2_length_distribution.pdf]

# Length Distribution

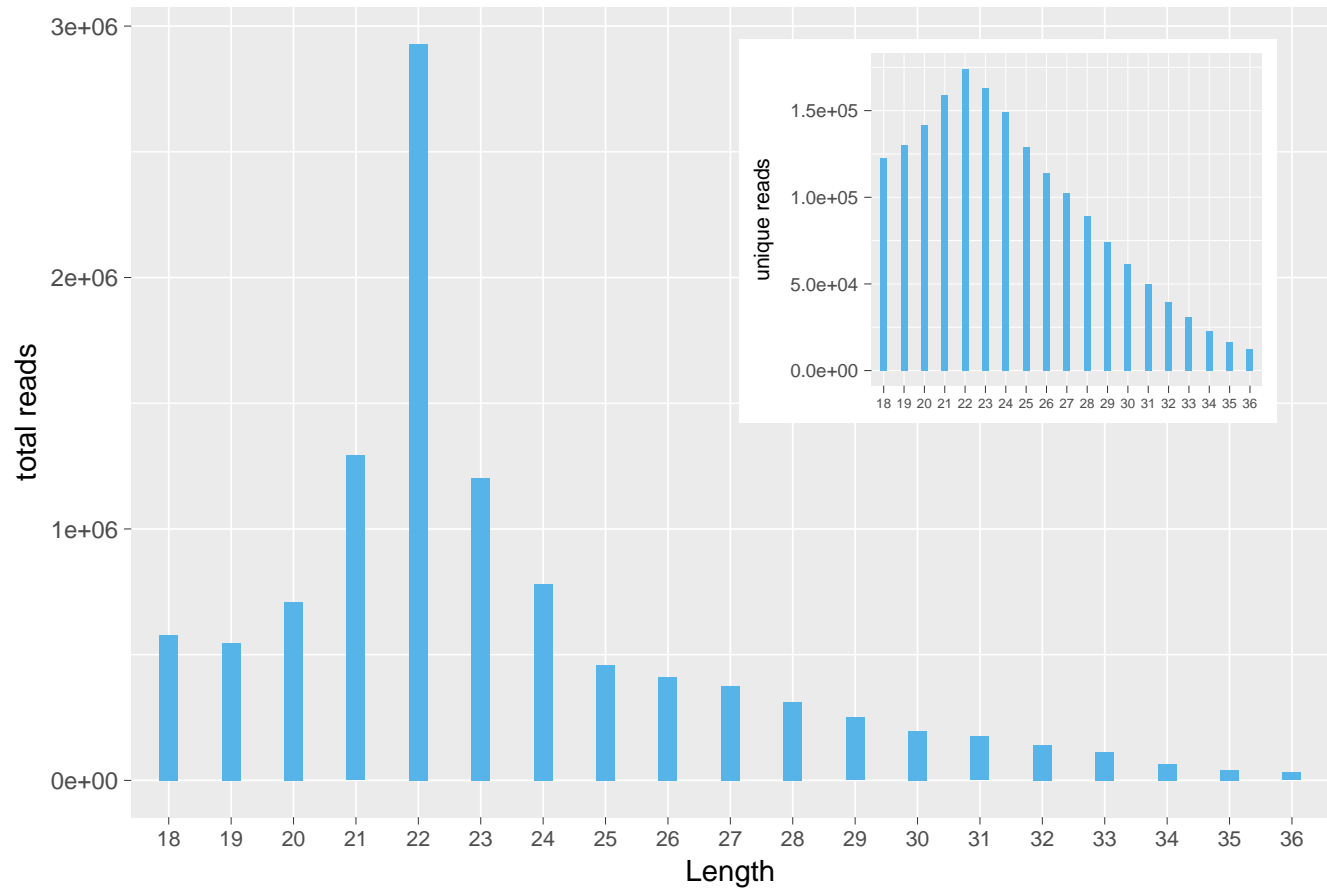

Supplement: Supplementary file 1 [file ijms-20-01041-s001.zip › supplement/Figure S1/con2-3_length_distribution.pdf]

# Length Distribution

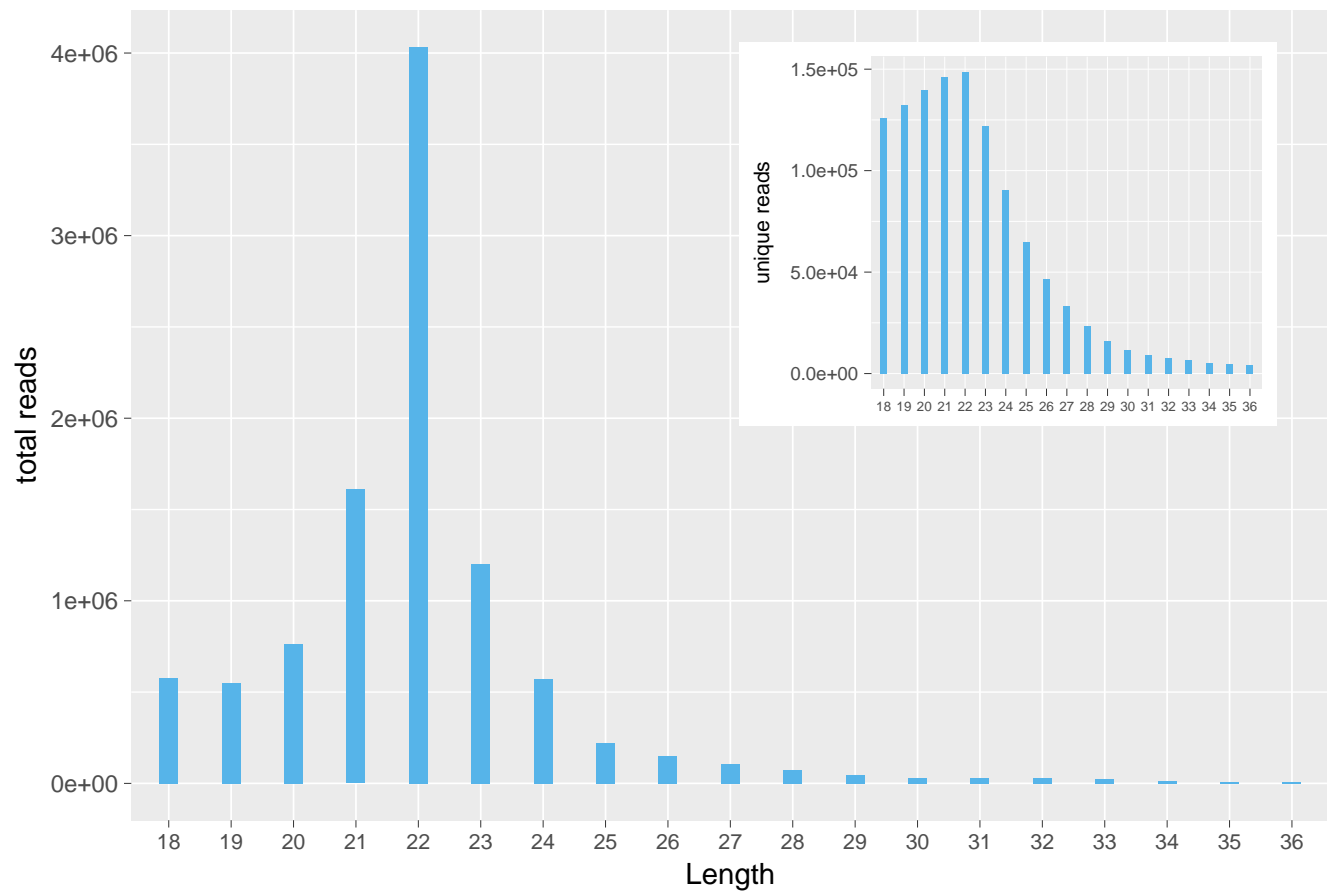

Supplement: Supplementary file 1 [file ijms-20-01041-s001.zip › supplement/Figure S1/con3-1_length_distribution.pdf]

# Length Distribution

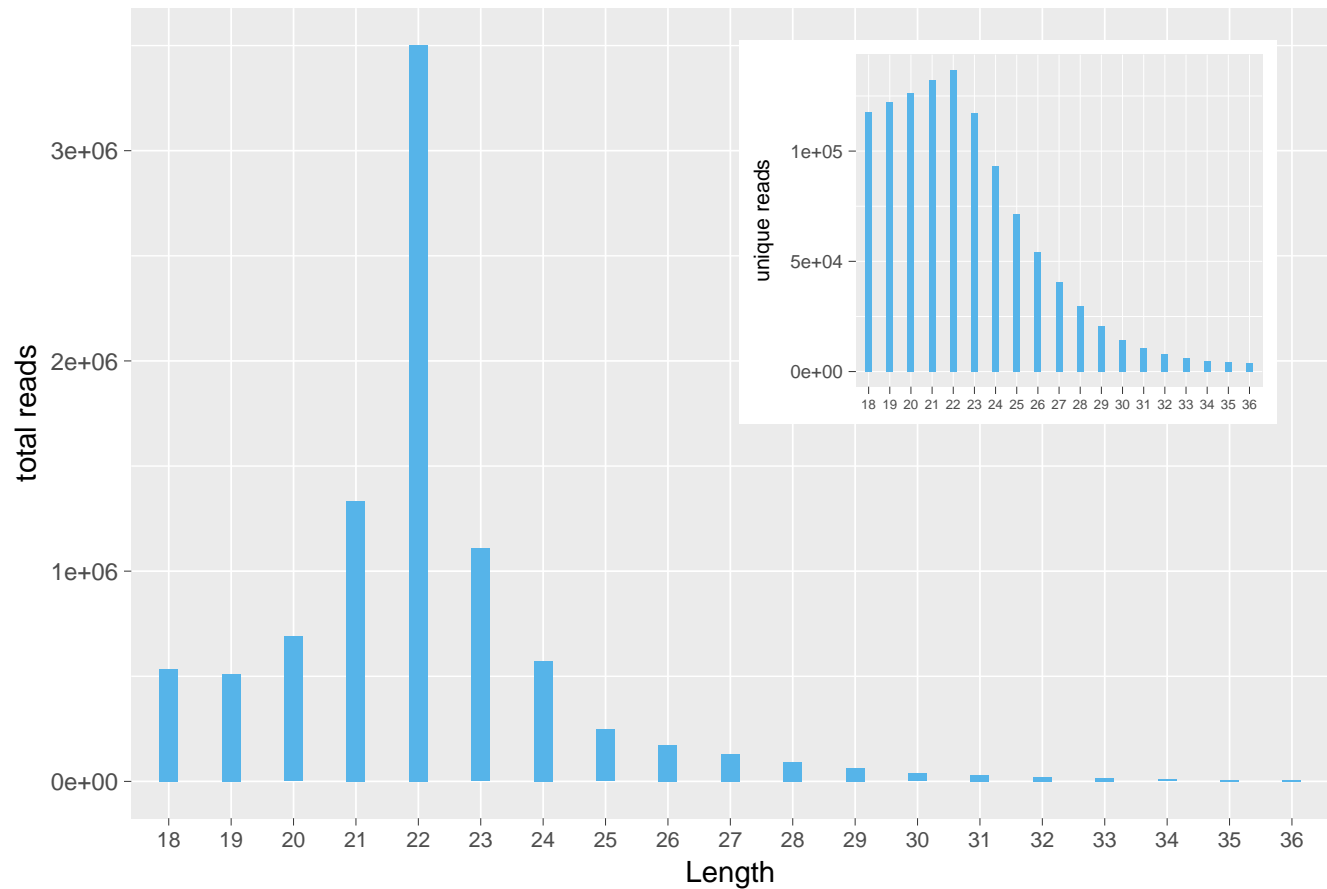

Supplement: Supplementary file 1 [file ijms-20-01041-s001.zip › supplement/Figure S1/con3-2_length_distribution.pdf]

# Length Distribution

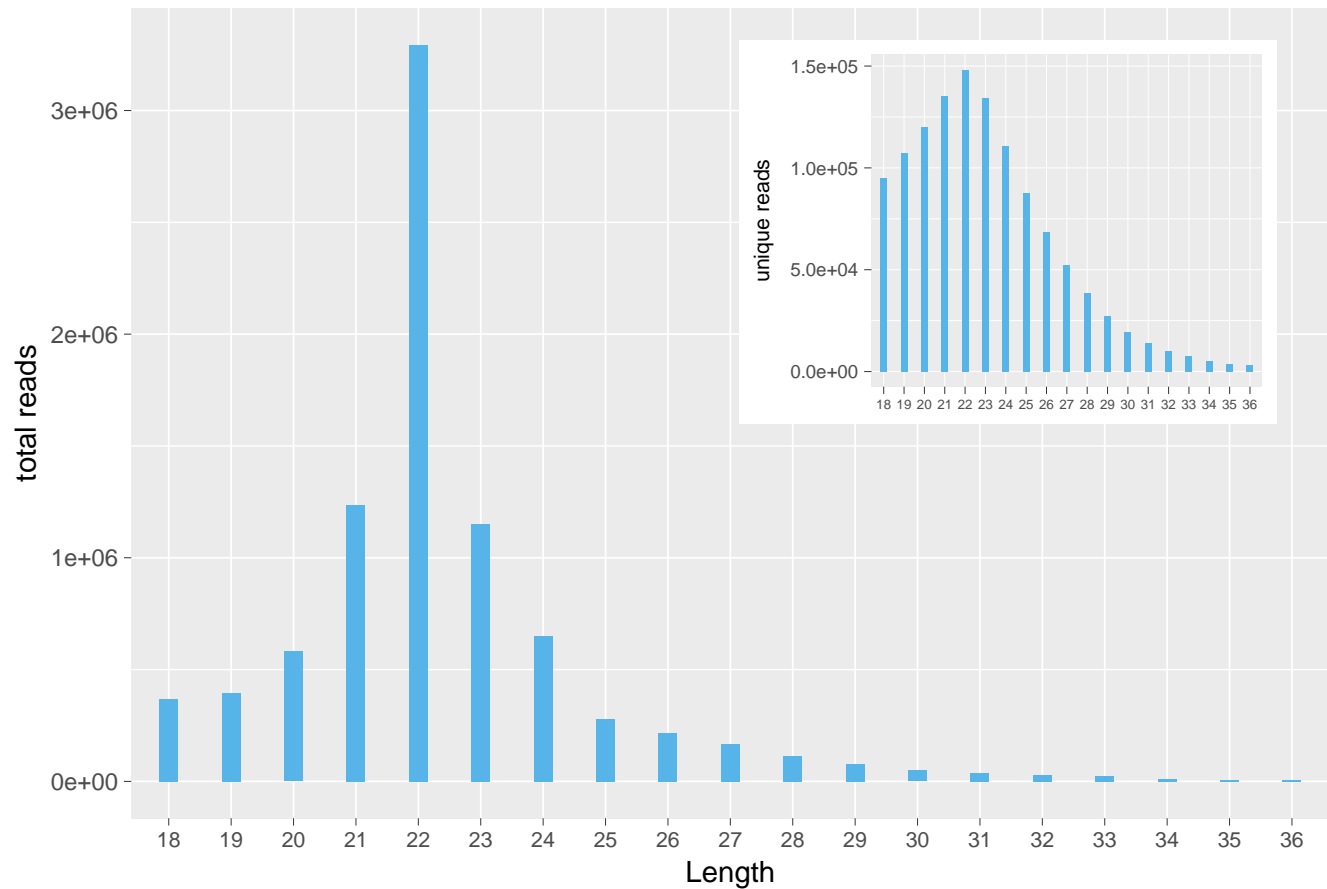

Supplement: Supplementary file 1 [file ijms-20-01041-s001.zip › supplement/Figure S1/con3-3_length_distribution.pdf]

# Length Distribution

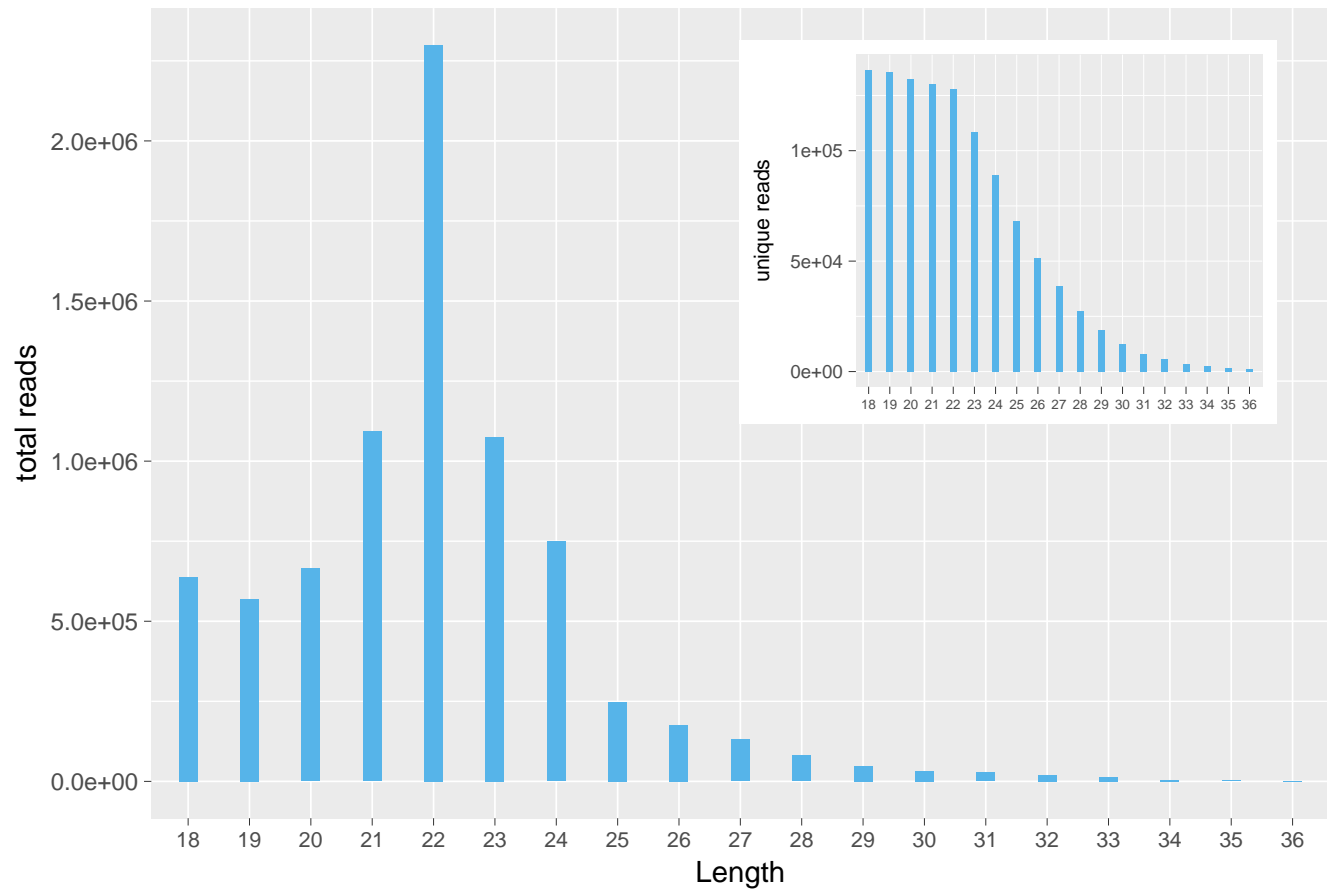

Supplement: Supplementary file 1 [file ijms-20-01041-s001.zip › supplement/Figure S1/inf1-2_length_distribution.pdf]

# Length Distribution

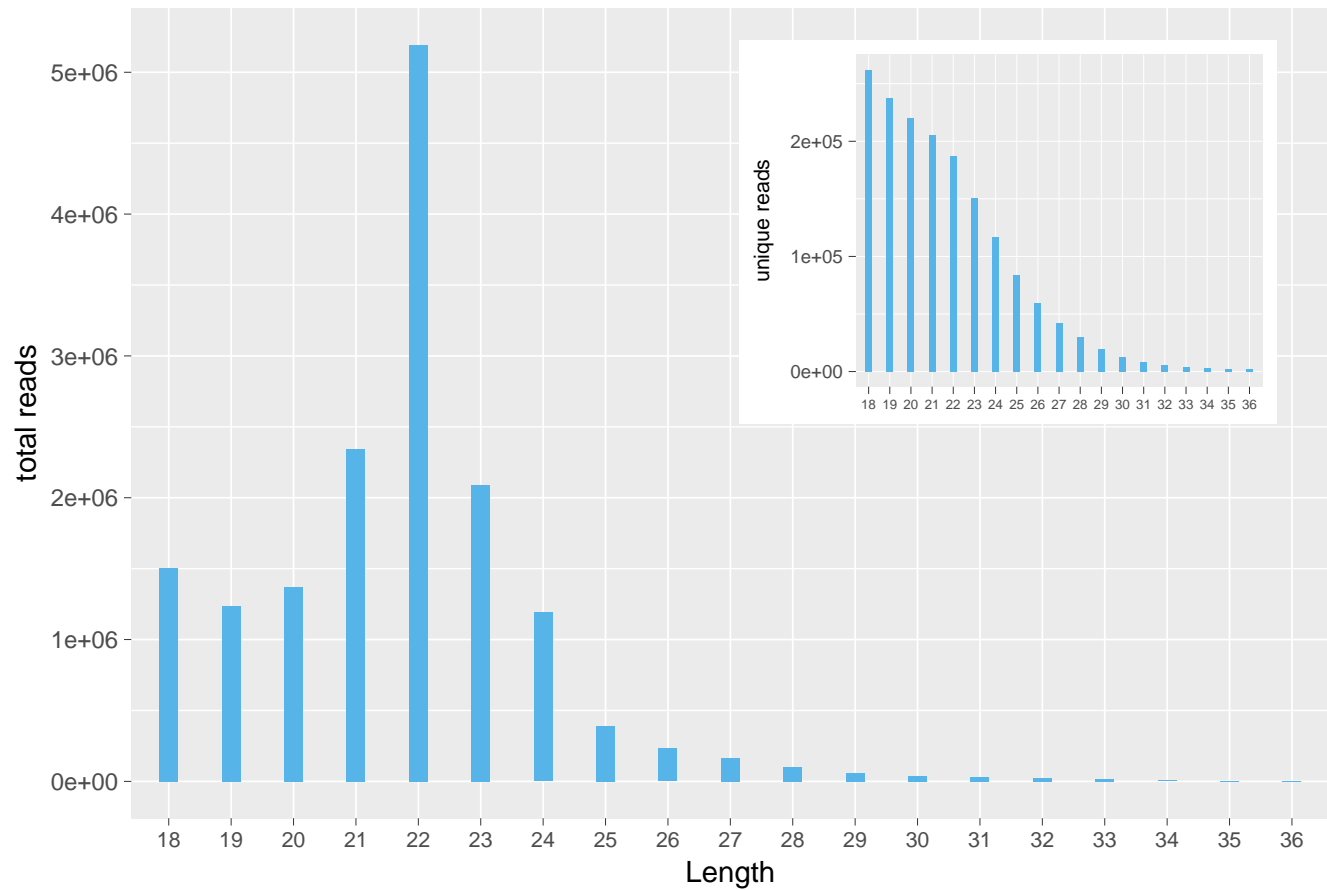

Supplement: Supplementary file 1 [file ijms-20-01041-s001.zip › supplement/Figure S1/inf1-3_length_distribution.pdf]

# Length Distribution

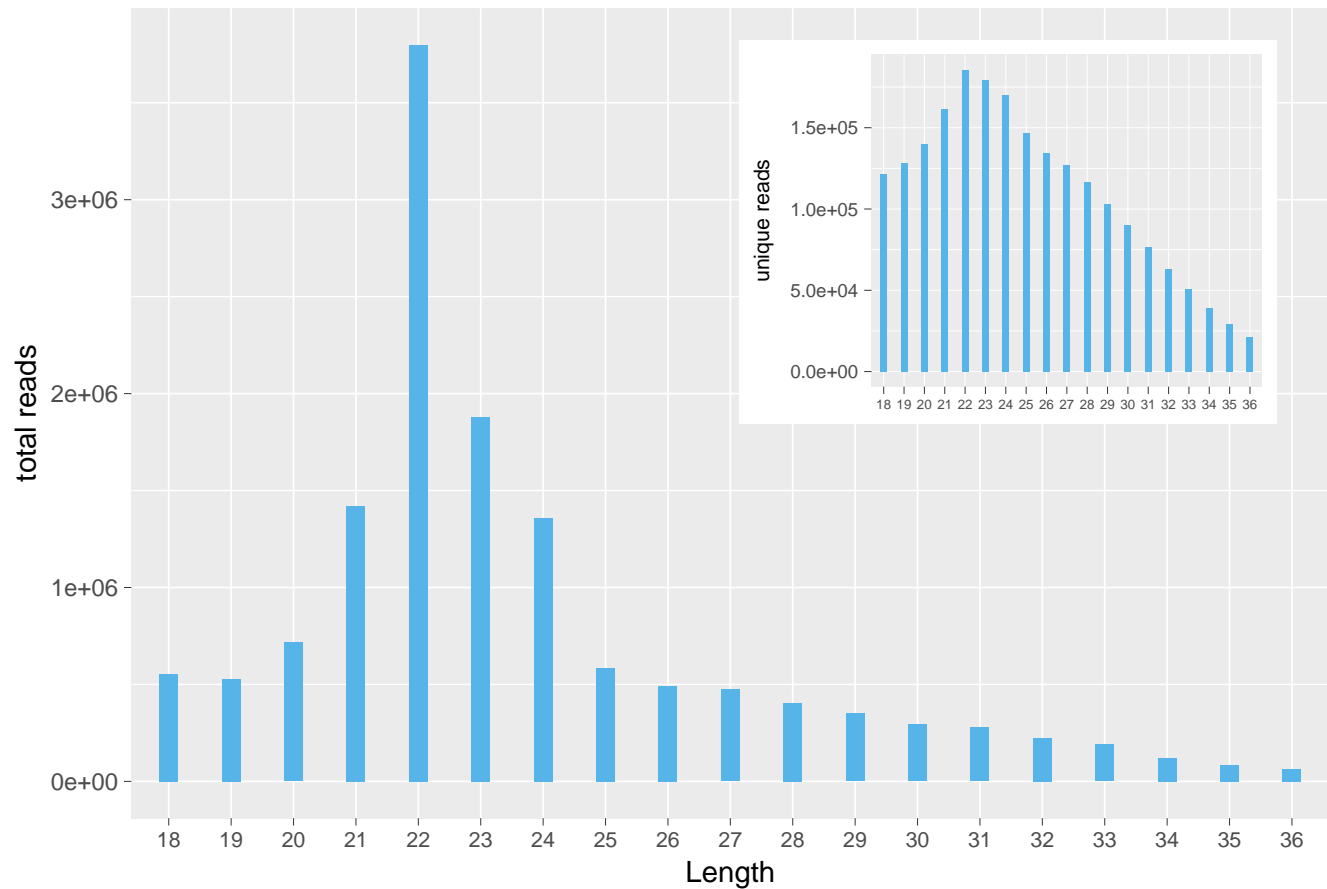

Supplement: Supplementary file 1 [file ijms-20-01041-s001.zip › supplement/Figure S1/inf2-1_length_distribution.pdf]

# Length Distribution

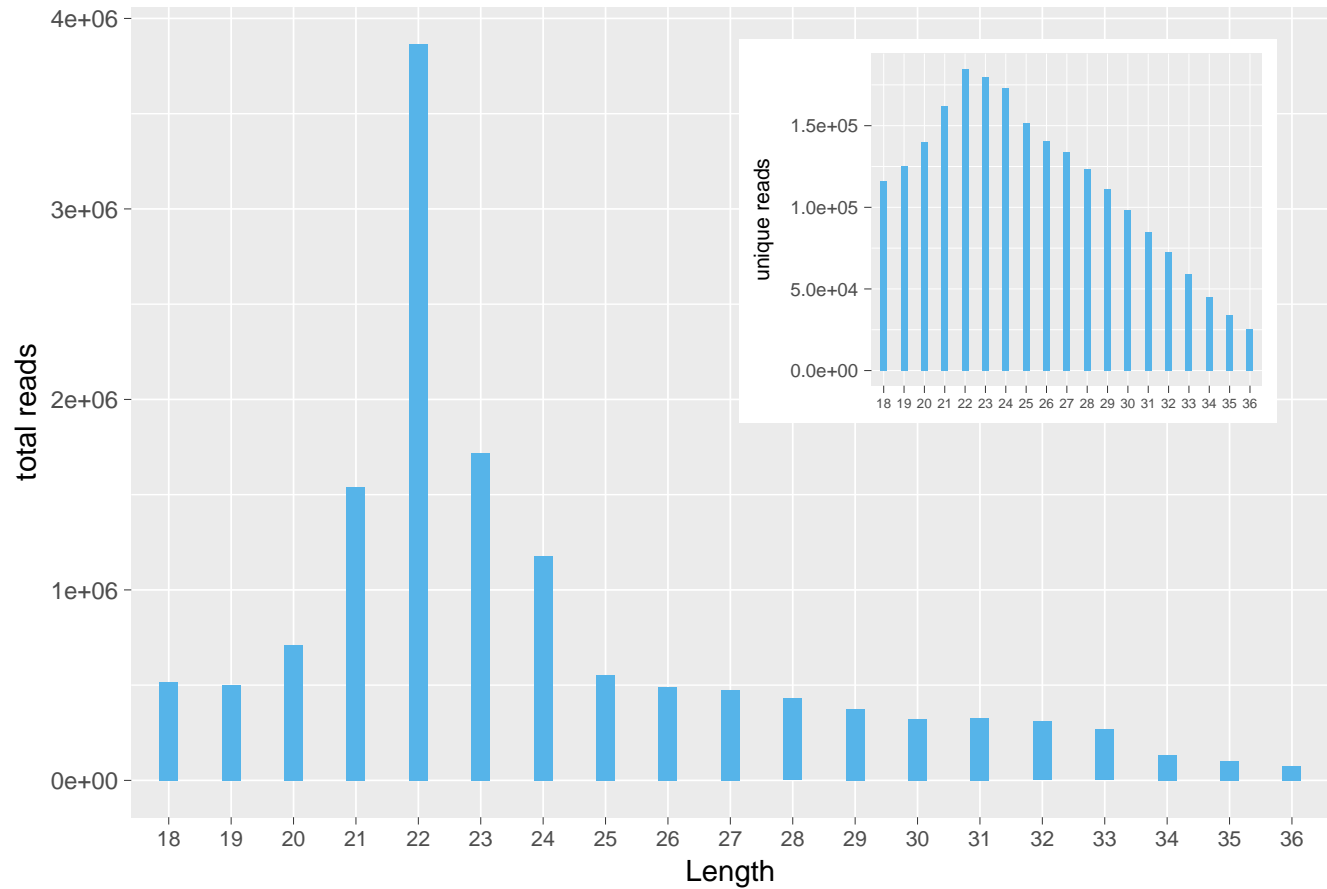

Supplement: Supplementary file 1 [file ijms-20-01041-s001.zip › supplement/Figure S1/inf2-2_length_distribution.pdf]

# Length Distribution

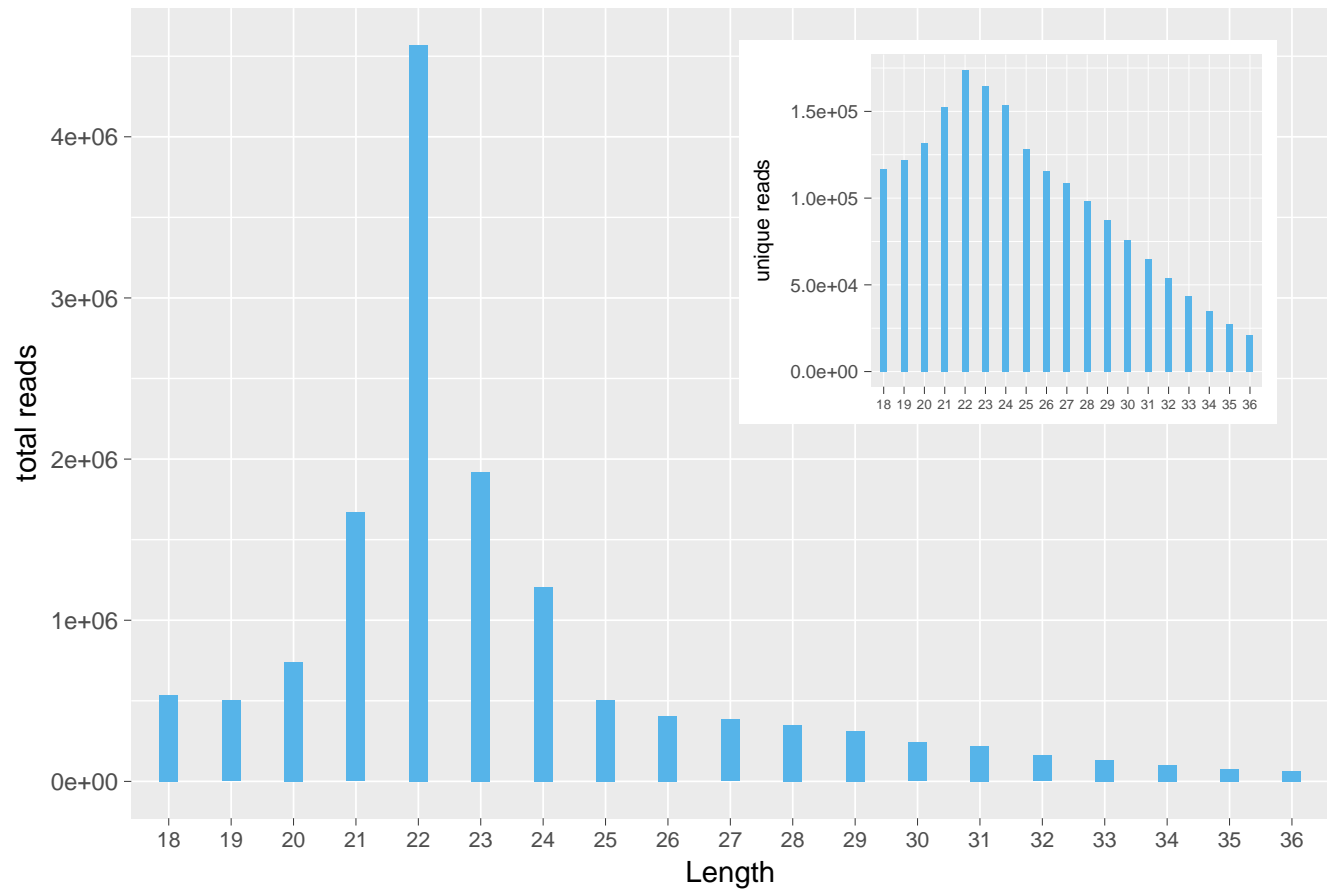

Supplement: Supplementary file 1 [file ijms-20-01041-s001.zip › supplement/Figure S1/inf2-3_length_distribution.pdf]

# Length Distribution

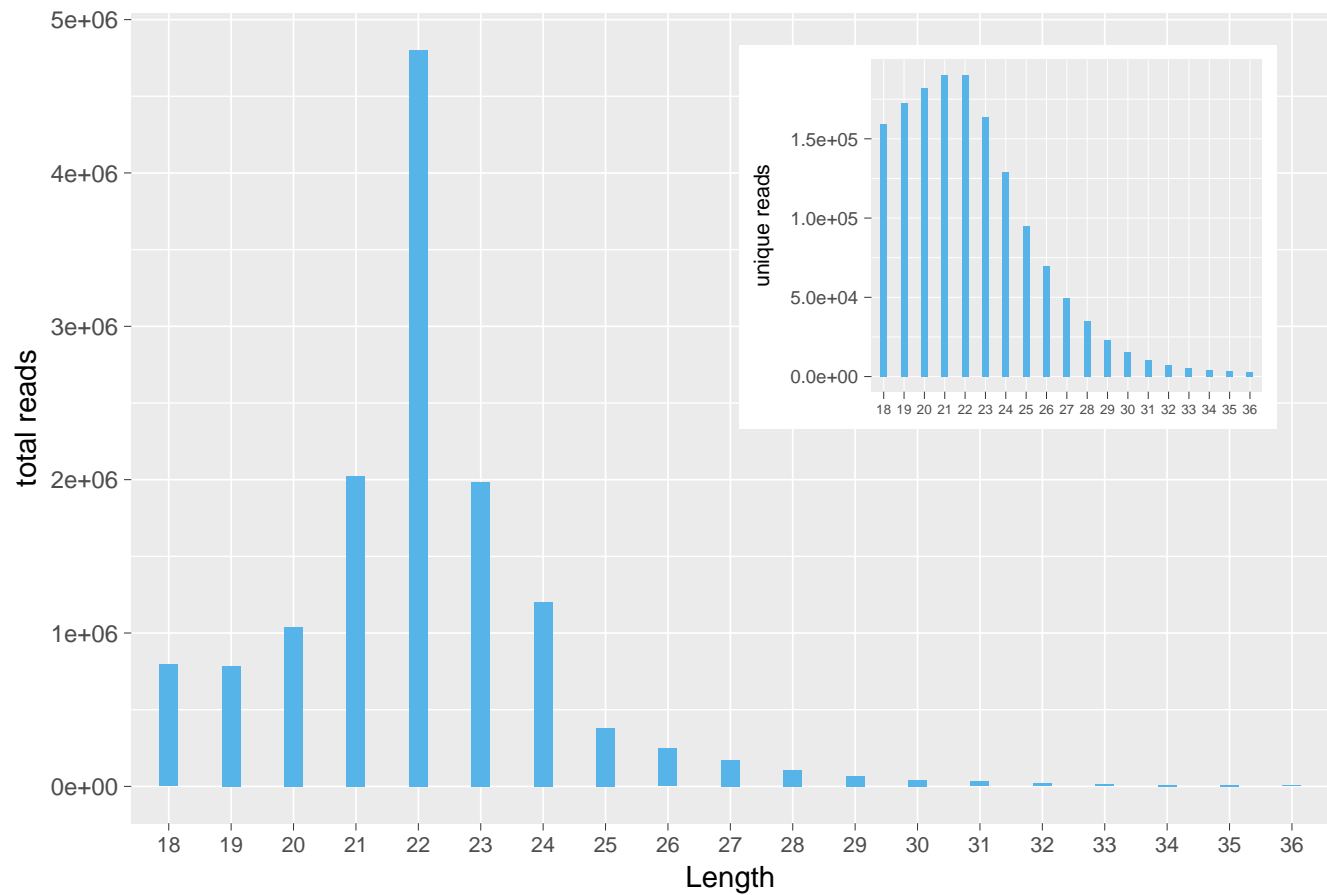

Supplement: Supplementary file 1 [file ijms-20-01041-s001.zip › supplement/Figure S1/inf3-1_length_distribution.pdf]

# Length Distribution

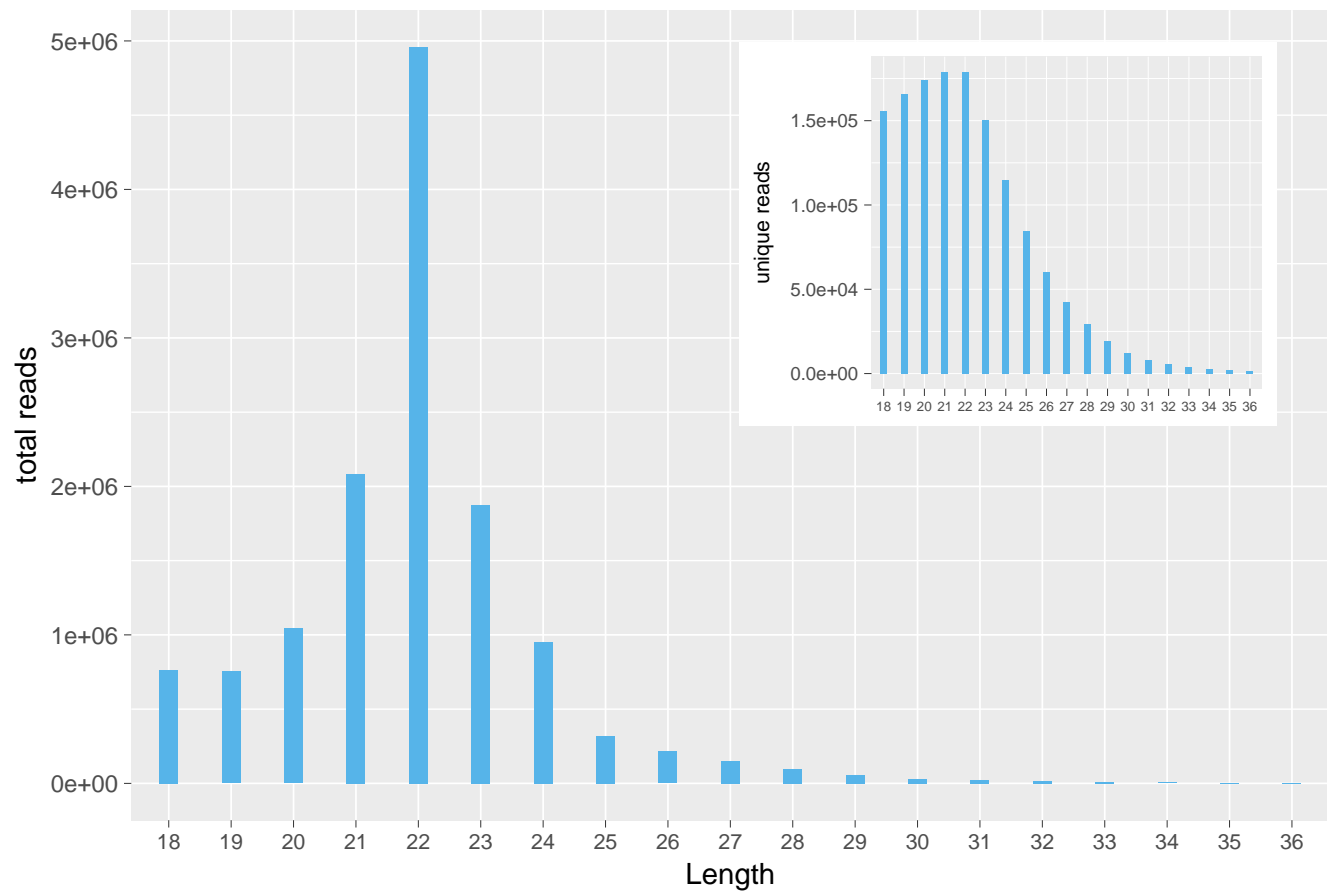

Supplement: Supplementary file 1 [file ijms-20-01041-s001.zip › supplement/Figure S1/inf3-2_length_distribution.pdf]

# Length Distribution

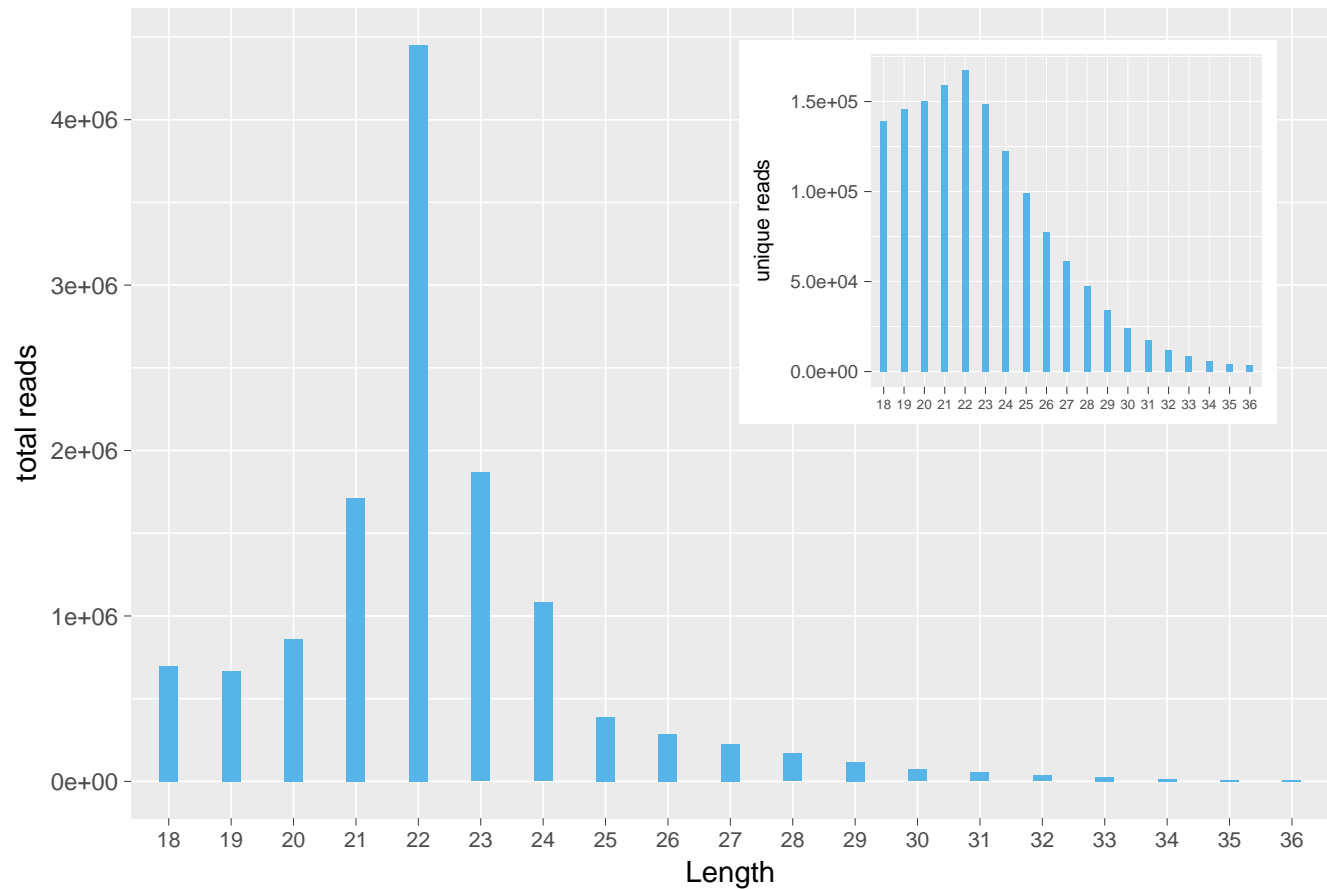

Supplement: Supplementary file 1 [file ijms-20-01041-s001.zip › supplement/Figure S1/inf3-3_length_distribution.pdf]

# Length Distribution

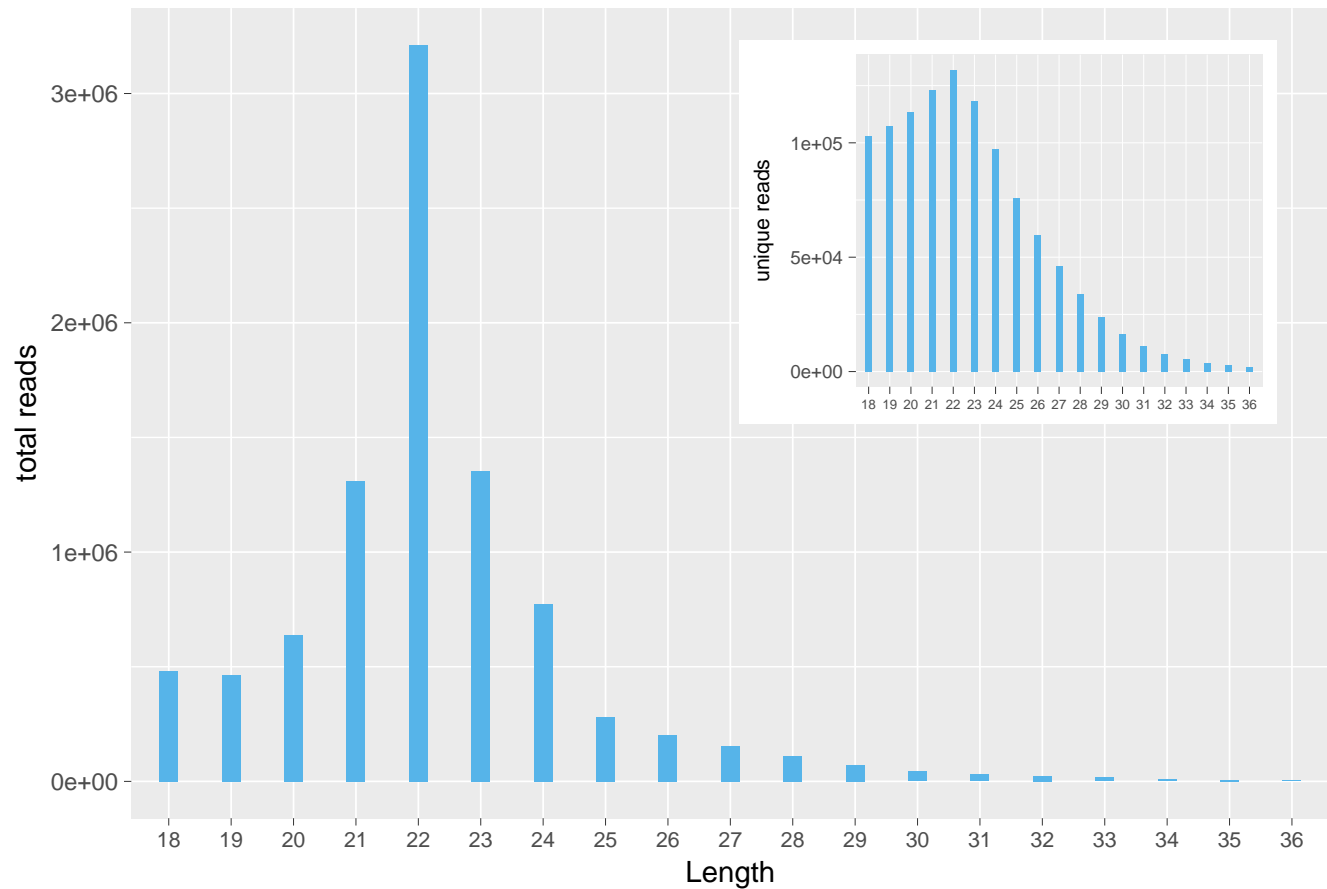

Supplement: Supplementary file 1 [file ijms-20-01041-s001.zip › supplement/Figure S1/inf3-4_length_distribution.pdf]
